# Similar Methanogenic Shift but Divergent Syntrophic Partners in Anaerobic Digesters Exposed to Direct versus Successive Ammonium Additions

**DOI:** 10.1128/Spectrum.00805-21

**Published:** 2021-10-06

**Authors:** Julie Hardy, Patricia Bonin, Adele Lazuka, Estelle Gonidec, Sophie Guasco, Corinne Valette, Sébastien Lacroix, Léa Cabrol

**Affiliations:** a MIO, Aix Marseille University, University of Toulon, CNRS, IRD, Marseille, France; b Scientific & Technological Expertise Department, Veolia, Maisons-Laffitte, France; c Instituto de Ecologia y Biodiversidad (IEB) Facultad de Ciencias, Universidad de Chile Las Palmeras, Nunoa, Santiago, Chile; University of Minnesota

**Keywords:** acetate, syntrophic, methanogen disturbance, adaptation, acclimation, ammonia, disturbance, methane, *Methanoculleus*, microbial diversity, perturbation, stepwise, stress adaptation, syntrophic acetate oxidation (SAO), syntrophs, volatile fatty acids (VFA)

## Abstract

During anaerobic digestion (AD) of protein-rich wastewater, ammonium (NH_4_^+^) is released by amino acid degradation. High NH_4_^+^ concentrations disturb the AD microbiome balance, leading to process impairments. The sensitivity of the AD microbiome to NH_4_^+^ and the inhibition threshold depend on multiple parameters, especially the previous microbial acclimation to ammonium stress. However, little is known about the effect of different NH_4_^+^ acclimation strategies on the differential expression of key active microbial taxa. Here, we applied NH_4_^+^ inputs of increasing intensity (from 1.7 to 15.2 g N-NH_4_^+^ liters^−1^) in batch assays fed with synthetic wastewater, according to two different strategies: (i) direct independent inputs at a unique target concentration and (ii) successive inputs in a stepwise manner. In both strategies, along the NH_4_^+^ gradient, the active methanogens shifted from acetoclastic *Methanosaeta* to *Methanosarcina* and eventually hydrogenotrophic *Methanoculleus*. Despite shorter latency times, the successive input modality led to lower methane production rate, lower soluble chemical oxygen demand (sCOD) removal efficiency, and lower half maximal inhibitory concentration, together with higher volatile fatty acid (VFA) accumulation, compared to the independent input modality. These differential performances were associated with a drastically distinct succession pattern of the active bacterial partners in both experiments. In particular, the direct exposure modality was characterized by a progressive enrichment of VFA producers (mainly *Tepidimicrobium*) and syntrophic VFA oxidizers (mainly *Syntrophaceticus*) with increasing NH_4_^+^ concentration, while the successive exposure modality was characterized by a more dynamic succession of VFA producers (mainly *Clostridium*, *Sporanaerobacter*, *Terrisporobacter*) and syntrophic VFA oxidizers (mainly *Tepidanaerobacter*, *Syntrophomonas*). These results bring relevant insights for improved process management through inoculum adaptation, bioaugmentation, or community-driven optimization.

**IMPORTANCE** Anaerobic digestion (AD) is an attractive biotechnological process for wastewater bioremediation and bioenergy production in the form of methane-rich biogas. However, AD can be inhibited by ammonium generated by protein-rich effluent, commonly found in agro-industrial activities. Insights in the microbial community composition and identification of AD key players are crucial for anticipating process impairments in response to ammonium stress. They can also help in defining an optimal microbiome adapted to high ammonium levels. Here, we compared two strategies for acclimation of AD microbiome to increasing ammonium concentration to better understand the effect of this stress on the methanogens and their bacterial partners. Our results suggest that long-term cumulative exposure to ammonia disrupted the AD microbiome more strongly than direct (independent) ammonium additions. We identified bioindicators with different NH_4_^+^ tolerance capacity among VFA producers and syntrophic VFA oxidizers.

## INTRODUCTION

Anaerobic digestion (AD) relies on the microbial degradation of organic matter and its conversion to methane (CH_4_), leading to reduced waste volume and energetically valuable biogas. Anaerobic digestion involves four metabolic steps performed by specific microbial groups. Briefly, hydrolytic and fermentative bacteria produce mainly short-chain fatty acids (SCFA, e.g., acetate, propionate, butyrate), dihydrogen (H_2_), and carbon dioxide (CO_2_) via hydrolysis, acidogenesis, and acetogenesis. In turn, acetate and H_2_/CO_2_ are converted into CH_4_ by, respectively, acetoclastic (e.g., *Methanosaetaceae*, *Methanosarcinaceae*) and hydrogenotrophic (e.g., *Methanomicrobiales*, *Methanobacteriales*) archaea. Acetate and other SCFA can also be oxidized to H_2_ by syntrophic acetate-oxidizing (SAO) bacteria. Although this reaction is thermodynamically unfavorable under standard conditions (ΔG_0_ = +105 kJ), it can occur via syntrophic interaction with hydrogenotrophic methanogens, enabling the maintenance of a sufficiently low H_2_ partial pressure for syntrophic acetate-oxidizing bacteria (SAOB) to thrive (1–3). The relative contribution of acetoclastic methanogenesis and SAO-coupled hydrogenotrophic methanogenesis in AD depends on many environmental conditions (4).

The AD microbiome balance can be affected by different inhibitors, leading to process instability and reduced biogas yield. Ammonia, released by the degradation of proteins and/or urea, usually leads to suboptimal AD performance when treating nitrogen-rich substrates, such as manure, livestock processing or textile industry wastewater, and food waste (especially meat-processing, seafood, and dairy industries), with ammonium levels ranging some tens of milligrams liter^−1^ to some thousands of milligrams liter^−1^ (5–7) (for examples, see Table S1). TAN (total ammonia nitrogen) is the sum of nitrogen in the form of nonionized free ammonia (FAN or NH_3_) and ammonium ion (NH_4_^+^), the equilibrium between both forms depending mainly on pH and temperature. Previous studies reported a high disparity in TAN inhibitory thresholds in AD, with 50% inhibitory concentration (IC_50_) ranging from 1.1 to 11.8 g TAN-N liter^−1^ (8–10). The variability of ammonium sensitivity or tolerance might be caused by different microbial community structures, coming from different inocula exposed to distinct history and acclimation strategy. Other operating parameters (such as temperature, substrate composition, organic loading rate, pH control, reactor configuration), which are not necessarily comparable between different studies, also have an important crossed influence on ammonium inhibition, resulting in high apparent variability of AD resistance capacity (10).

The inhibition of methane production in response to ammonium can result from (i) a direct inhibitory effect on methanogens and other community members (e.g., through cell diffusion, pH modification, and proton imbalance) (7) and/or (ii) an indirect effect, in which the lower VFA consumption by methanogens leads to VFA accumulation, thereby enhancing inhibition of methanogens and other AD members, in a negative feedback loop (11). Indeed, in addition to methanogens, VFA are well-known inhibitors of SAOB, acetogens, and hydrolytic bacteria (12, 13) and strongly influence methanogenic communities (14–16). Even if syntrophic bacteria tolerate high VFA and ammonia levels better than methanogens (their growth being promoted at moderate VFA levels), they are also eventually inhibited at high VFA levels (17, 18).

Under ammonia stress, several studies report that acetoclastic methanogens are more severely inhibited than hydrogenotrophic methanogens and that SAOB are promoted (4, 7, 19, 20). However, the differential tolerance and adaptation capacities of individual, active (RNA-based) AD species to ammonia remains poorly understood. In particular, the possible acclimation to ammonia through different exposure modes has been rarely evaluated (21, 22), despite its interest as a microbial resource management strategy enhancing ammonia resistance. Since multiple taxa are often able to carry out the same function in AD, a flexible community structure could increase process stability toward ammonium disturbances via functional redundancy with differential ammonia tolerance capacities (23). Moreover, the variability of the feeding pattern can enhance AD functional stability when exposed to further shocks (24).

Particularly sensitive taxa can be considered early bioindicators of process impairment. In addition, the identification of highly active keystone species playing a central role under AD disturbance is of paramount importance to maintain functional stability through process management, either by applying optimal operating conditions favoring their growth or by bioaugmentation (20, 25, 26).

**FIG 1:**
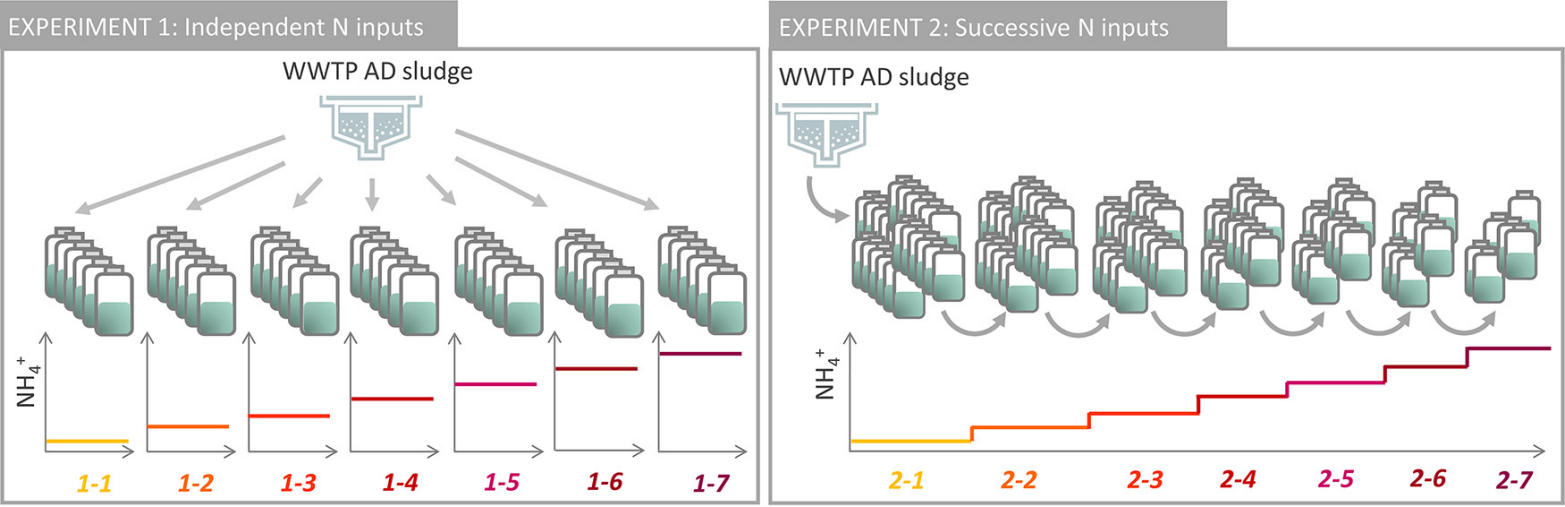
Experimental setup for experiment 1 (independent ammonium additions) and experiment 2 (successive ammonium additions). The N-NH_4_^+^ concentration corresponding to each condition is given in Table 1. At each NH_4_^+^ level, three replicated vials were sacrificed for biochemical and microbiological analysis at the time of maximal methanogenic activity, while the others were maintained (experiment 1) or transferred to the next level (experiment 2).

Due to the importance of historical contingencies in shaping microbial community structure, we assumed that different adaptation strategies of AD microbiome to ammonium stress would result in the enrichment of different tolerant taxa, leading to different inhibition levels. The objectives of the current study were (i) to investigate the effect of elevated ammonium stress on both the functional and structural responses of AD microbiome in batch assays, with a special attention to the active community, and (ii) to compare these responses according to the stress exposure modality. Namely, in one experiment, each microbial community was exposed to a direct (and unique) high NH_4_^+^ concentration (in the range 1.7 to 15.2 g N-NH_4_^+^ liter^−1^), while in the other experiment the microbial community was successively exposed to stepwise increasing NH_4_^+^ concentrations in the same range (Fig. 1).

## RESULTS

### Effect of ammonium inputs on biomethane potential (BMP) performances.

**FIG 2:**
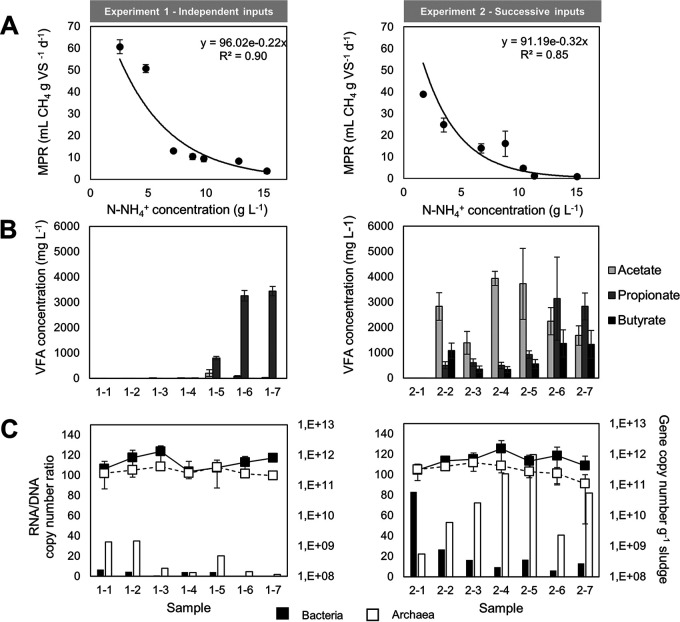
(A) Methane production rate (MPR) in experiment 1 (independent N inputs) and experiment 2 (successive N inputs) for each ammonium condition. MPR was calculated as the maximal slope of methane production kinetics along time (Fig. S1). Mean and standard deviation were calculated on 6 and 6 to 24 replicates for experiments 1 and 2, respectively. (B) Volatile fatty acid (VFA) concentration in experiments 1 and 2 (the N-NH_4_^+^ concentration corresponding to each sample number is given in Table 1). VFA concentrations were measured at the time of maximal methanogenic activity (end of exponential phase), and mean and standard deviation were calculated from triplicated incubation vials. (C) Abundances of present (DNA-based) bacteria and archaea for each N-NH_4_^+^ level in experiment 1 and 2, represented as dots. Mean and standard deviations were calculated from technical duplicates on biological duplicates (i.e., *n* = 4). For each condition, the transcript-to-gene ratio (cDNA/DNA), indicative of active expression level, is represented as bars.

The highest methane production rates (MPR; 50 ± 15 ml CH_4_ g^−1^ volatile solids [VS] day^−1^) were observed in the control at the lowest ammonium concentration (Table 1; Fig. S2). Overall, the MPR rapidly decreased when N-NH_4_^+^ concentration increased, irrespective of N-input modalities (Fig. 2A). The magnitude of inhibition was stronger for experiment 2 than for experiment 1, with half maximal inhibitory concentration (IC_50_) of 3.30 and 2.19 g N-NH_4_^+^ liter^−1^, respectively. The sCOD removal efficiency was always higher in experiment 1 than in experiment 2. It also decreased with ammonium concentration, more abruptly in experiment 2 (Table 1), just as the methane yield (Kruskal-Wallis [KW], *n* = 7, *P* = 0.034).

**TABLE 1:** Summary of experimental conditions and resulting functional performance in batch assays of biomethane production exposed to independent ammonium inputs (experiment 1) and successive ammonium inputs (experiment 2)^*a*^

Experiment	Condition	N-NH_4_^+^ concentration (g liter^−1^)	Latency phase (days)	MPR (ml CH_4_ g^−1^ VS days^−1^)	CH_4_ yield (ml CH_4_ g^−1^ COD)	Residual COD (g liter^−1^)	COD removal efficiency (%)
Experiment 1 (independent inputs)	1-1	1.7 (0.1)	0 (0)	60.6 (3.2)	299.6 (20.7)	0.2 (0.1)	99.1 (0.5)
	1-2	4.8 (0.1)	0 (0)	50.7 (1.8)	310.8 (21.1)	1.0 (0.3)	94.3 (1.5)
	1-3	7.2 (0.3)	16.7 (1.8)	12.9 (0.8)	331.1 (23.2)	4.6 (0.7)	73.9 (4.0)
	1-4	8.9 (0.2)	20.8 (3.1)	10.3 (1.3)	269.1 (24.5)	6.0 (0.2)	65.6 (1.1)
	1-5	9.8 (0.4)	20.8 (2.6)	9.3 (1.3)	238.6 (33.2)	9.4 (0.6)	46.6 (3.4)
	1-6	12.8 (0.6)	20.8 (4.2)	8.1 (0.7)	179.6 (6.5)	11.6 (0.8)	33.9 (4.4)
	1-7	15.3 (0.9)	66.7 (4.6)	3.6 (0.9)	201.8 (17.5)	12.2 (1.6)	30.1 (9.1)
Experiment 2 (successive inputs)	2-1	1.7 (0.1)	0 (0.0)	38.4 (2.6)	309.9 (32.4)	1.7 (0.1)	90.4 (0.0)
	2-2	3.5 (0.3)	1.0 (0.5)	25.1 (3.6)	144.7 (26.4)	7.7 (1.1)	56.2 (6.2)
	2-3	6.7 (1.4)	1.0 (0.5)	14.5 (2.5)	223.4 (31.8)	6.4 (2.5)	55.5 (9.8)
	2-4	8.8 (2.1)	2.1 (1.0)	19.0 (4.9)	171.5 (77.6)	12.9 (3.8)	26.2 (21.5)
	2-5	10.4 (0.8)	3.1 (1.0)	4.6 (1.1)	92.3 (28.1)	14.1 (3.3)	19.7 (18.6)
	2-6	11.3 (2.0)	3.8 (1.5)	1.0 (0.3)	17.5 (2.6)	14.7 (3.8)	16.0 (21.5)
	2-7	15.1 (0.3)	5.2 (1.5)	1.7 (0.3)	18.8 (8.1)	14.9 (2.0)	15.0 (11.3)

a MPR, methane production rate. All means and standard deviations (in parenthesis) were calculated from triplicated incubation vials.

The effect of ammonium concentration on the lag phase was highly dependent on the experiment type. In the independent experiment, the lag phase strongly increased from 0 days in the control to 20.8 ± 3.3 days for N-NH_4_^+^ concentrations between 8.9 and 12.8 g N-NH_4_^+^ liter^−1^ and finally reached 66.7 ± 4.6 days at 15.3 g N-NH_4_^+^ liter^−1^. In contrast, under successive N-NH_4_^+^ inputs, the lag phase was much shorter (0 to 5.2 ± 1.5 days) and independent of the ammonium concentration (KW, *n* = 14, *P* = 0.448).

The VFA profiles were significantly affected by ammonium concentration (KW, *n* = 42, *P* = 0.0028 [<0.05]) and input modality (KW, *n* = 42, *P* = 0.021 [<0.05]) (Fig. 2B). During the independent experiment, butyrate was not detected, acetate did not accumulate (<203 ± 131 mg liter^−1^), and propionate accumulation began only from 9.8 g N-NH_4_^+^ liter^−1^ (level 1-5), reaching 3,443 ± 181 mg liter^−1^ at the highest ammonium level. In contrast, in the successive experiment, acetate accumulation started as soon as 3.5 g N-NH_4_^+^ liter^−1^ (level 2-2) and maintained around 2,633 ± 1,052 mg liter^−1^ over the whole NH_4_^+^ range. Propionate and butyrate concentrations were always detected (around 612 ± 266 mg liter^−1^ up to 10.4 g N-NH_4_^+^ liter^−1^) and particularly increased at the highest N-NH_4_^+^ concentration, reaching, respectively, 2,983 ± 216 and 1,345 ± 21 mg liter^−1^.

### Effect of ammonium inputs on microbial abundances.

The absolute abundances of bacterial and archaeal 16S rRNA genes were not drastically affected by the N-NH_4_^+^ level or the modality of N-NH_4_^+^ inputs ([6.8 ± 3.6] × 10^11^ and [3.1 ± 1.1] × 10^11^ 16S rRNA gene copies per gram of sludge, respectively; Fig. 2C). The N-NH_4_^+^ input scenarios had a strong effect on the 16S rRNA transcript/gene ratio, which is a proxy of microbial activity. Under independent NH_4_^+^ inputs, the 16S rRNA transcript/gene ratio was always relatively low for bacteria (around 3.0 ± 2.6), while it was clearly higher for archaea (33.9 to 4.7, with a slight tendency to decrease with NH_4_^+^ concentration). Under successive NH_4_^+^ inputs, the bacterial activity indicator strongly decreased from 83.2 to 6.2 when N-NH_4_^+^ increased. In contrast, the archaeal activity indicator first increased with ammonium concentration (activation) to reach its maximal transcript level (119.5) at 10.4 g N-NH_4_^+^ liter^−1^ before decreasing at the highest ammonium levels. For both archaea and bacteria, the relative expression level was always higher in experiment 2 than in experiment 1 (by a factor of 24 on average, ranging from 1 to 160) at the same corresponding ammonium level (Fig. 2C).

### Effect of ammonium inputs on microbial diversity.

The magnitude of diversity loss induced by NH_4_^+^ increase was stronger for the successive shocks than for the independent shocks (analysis of variance [ANOVA], *P* < 0.002) (Fig. S4). In experiment 1, the observed richness (at the DNA level) significantly increased with increasing N-NH_4_^+^ concentrations (ANOVA, *P* = 0.0004; Spearman correlation *R* = 0.9, *P* < 10^−4^), whereas at the RNA level the observed richness and Shannon diversity index slightly decreased with NH_4_^+^ concentration (Spearman *R* = −0.84 and −0.79, respectively; *P* < 0.0008). In experiment 2, richness and Shannon indexes were significantly negatively correlated with NH_4_^+^ concentration for present (Spearman *R* = −0.94 and −0.97, respectively, *P* < 10^−4^) and active (Spearman *R* = −0.96 and −0.76, respectively, *P* < 0.0026) communities. In experiment 1, the diversity was stable at low NH_4_^+^ concentrations and started to be affected by NH_4_^+^ concentrations above 9.8 g N-NH_4_^+^ liter^−1^, while in experiment 2, the effect of NH_4_^+^ concentration on diversity was stronger at the beginning and alleviated above 10.4 g N-NH_4_^+^ liter^−1^.

### Taxonomic composition at the phylum level.

**FIG 3:**
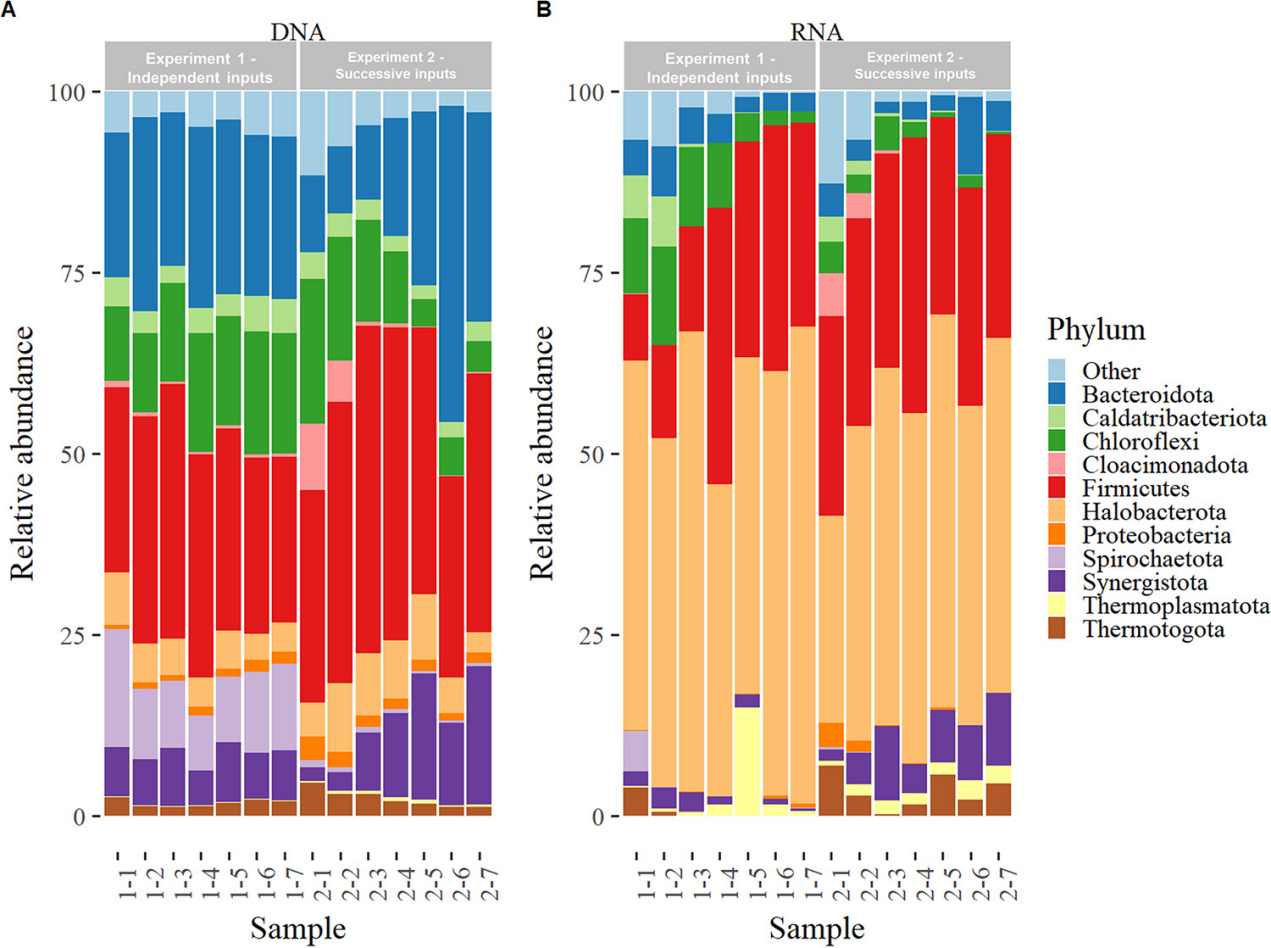
Taxonomic affiliation of the 11 most abundant phyla, obtained from the 16S rRNA gene (DNA) and transcripts (cDNA) sequences. The group “Other” contains all phyla with relative abundance lower than 0.5% of the community on average over all samples.

At the phylum level, the present (DNA-based) community was rather similar between independent- and successive-shock strategies, dominated by *Firmicutes* (representing 32.5% ± 6.9% of the community in both experiments), followed by *Bacteroidota* (21.8 ± 8.9%) and *Chloroflexi* (12.4% ± 5.2%) (Fig. 3). Contrastingly between both strategies, the NH_4_^+^ concentration increase in experiment 2 was associated with a decrease of *Chloroflexi* (from 20.0 to 3.7% of the community) and an increase of *Synergistota* (from 1.8 to 19.2%) and *Bacteroidota* (from 9.3 to 43.7%) abundances, while these three phyla stayed stable in experiment 1 (15% of relative standard deviation, on average). Archaea represented 6.4% ± 2.5% of the present community in both experiments, largely dominated by *Halobacterota* (representing 92.5% ± 4.3% of archaea).

The most drastic change of community composition occurred between the present and active communities, with 82% ± 10% (experiment 1) and 66% ± 6% (experiment 2) pairwise dissimilarity on average between DNA- and RNA-based community for each NH_4_^+^ condition. This dissimilarity increased with NH_4_^+^ concentration (*R*^2^ of 0.95 and 0.74 in experiments 1 and 2, respectively), revealing the enhanced divergence associated with more drastic experimental conditions. Archaea represented 52.4% ± 9.5% of the active prokaryotes in both experiments and were dominated by *Halobacterota* (representing 94.5% ± 0.1% of archaea). Among them, *Methanoculleus* was always the dominant active methanogen (Fig. S5). The second most abundant active phylum was *Firmicutes*. Its abundance increased with NH_4_^+^ concentration in experiment 1 (from 9.2 to 38.2% of the active community), while it stayed stable in experiment 2 (29.9% ± 3.8% of the active community). At the phylum level, the increase of NH_4_^+^ concentration was associated with a decrease of *Chloroflexi* and *Bacteroidota* proportions in the active community, especially in experiment 1, and a slight increase of active *Synergistota*, observed exclusively in experiment 2 (6.5% ± 3.2% of the active community).

### Overall variations of the community structure.

**FIG 4:**
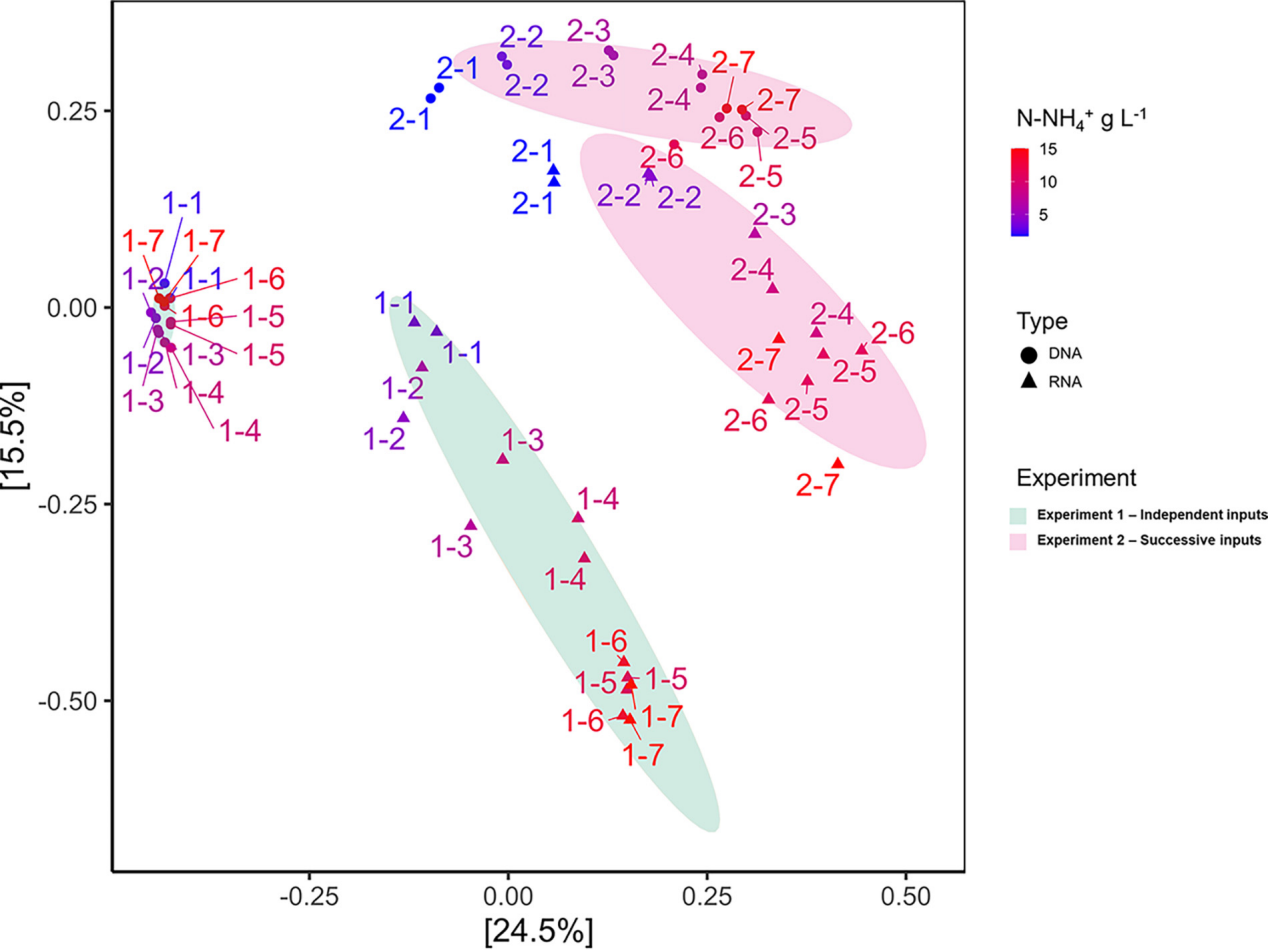
Principal coordinate analysis (PCoA) ordination of the present (DNA-based) and active (RNA-based) microbial communities from experiment 1 (independent N inputs) and experiment 2 (successive N inputs). Significant samples’ clustering was verified by nonparametric PERMANOVA (*adonis* function, 99 permutations, *P* < 0.001) and represented by ellipses built at 80% confidence interval level. All replicates are shown.

The structure of present and active communities was evaluated by a PCoA ordination, accounting for 40% of the total variance on the first two axes (Fig. 4). There was a high similarity between duplicated vials, higher for present (21% ± 9% pairwise Bray-Curtis [BC] dissimilarity on average) than for active (42% ± 20% pairwise BC dissimilarity on average) community, validating the robustness of our methodology. Despite similarities at the phylum level, the analysis at the amplicon sequence variant (ASV) level revealed strong divergence between both experiments (83% ± 6% pairwise dissimilarity on average between independent and successive input strategies). Based on variation partition analysis, the samples first clustered according to the targeted fraction of the community (present versus active), explaining 12.5% of the total variance, then according to the NH_4_^+^ input modality, explaining 12.0% of the variance, and finally according to the NH_4_^+^ concentration, accounting for 8.4% of the variance. This clustering was significantly supported by permutational multivariate analysis of variance (PERMANOVA; *P*< 0.001). The DNA-based community structure was more dynamic in experiment 2 than in experiment 1 (55% ± 18% versus 29% ± 8% pairwise dissimilarity on average, respectively).

### Relationships between active microbial communities and environmental variables.

**FIG 5:**
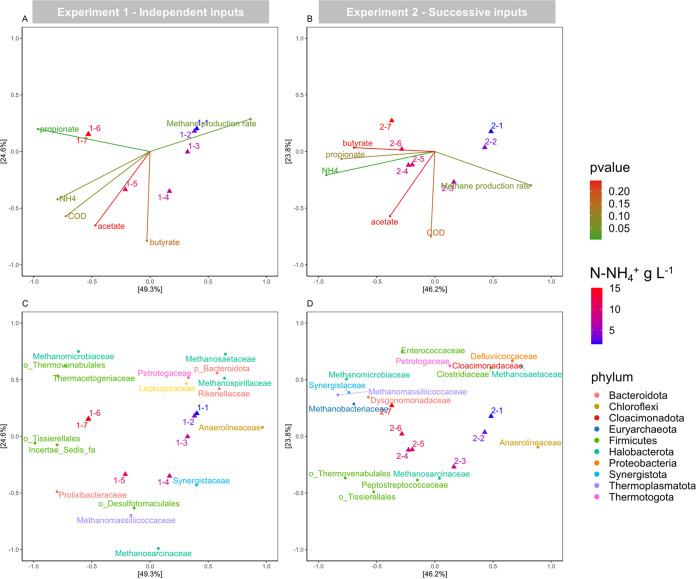
Principal coordinate analysis (PCoA) of the active microbial community in experiment 1 (independent ammonium inputs, panels A and C) and experiment 2 (successive ammonium inputs, panels B and D) based on Bray-Curtis dissimilarities. For each sample, the input ammonium concentration is indicated by the symbol color gradient. Duplicates are aggregated by the mean. (A and B) Correlation of the ordination with environmental and functioning variables (significance tested by *envfit* function, *P* value represented by the arrow color gradient). (C and D) Representation of the 50 most abundant ASVs from the active community of each experiment, aggregated at the family level and colored by phylum.

The relationship between active microbial communities and physicochemical variables was investigated by PCoA for each experiment separately. The ordinations accounted for 73.9% and 70.0% of the variance in experiments 1 and 2, respectively (Fig. 5).

Ammonium concentration was the major significant driver of the active community structure in both experiments. In experiment 1, the active community structure was also significantly associated with propionate concentration and residual sCOD, both increasing with NH_4_^+^ concentration (Fig. 5A). The active community structure was not significantly correlated with VFA concentrations in experiment 2, probably due to the sustainably high VFA level along the NH_4_^+^ gradient, without a significant evolution pattern (Fig. 5B). The PCoA ordinations revealed a clear succession of the most abundant active families along the ammonium gradient. The succession of methanogens was highly similar in both NH_4_^+^ input modalities. *Methanosaetaceae* was discriminant of lower NH_4_^+^ and VFA concentrations, whereas *Methanosarcinaceae* and *Methanomicrobiaceae* were the major contributors of intermediary and high ammonium conditions, respectively (Fig. 5C and D). The tipping point, i.e., the NH_4_^+^ threshold inducing the shift between the different methanogenic families, depended on the ammonium supply modality (10.4 and 6.7 g N-NH_4_^+^ liter^−1^ for experiments 1 and 2). The bacterial succession pattern was markedly distinct according to the ammonium input modality. The *Anaerolineaceae* family was characteristic of low ammonium levels in both experiments, but the highest ammonium levels were characterized by remarkably different families within the *Firmicutes* phylum. The identification and functional inference of enriched bacterial members will be further explored in the next sections.

### Dynamics of differentially expressed genera within active *Halobacterota* and *Firmicutes* in response to ammonia.

**FIG 6:**
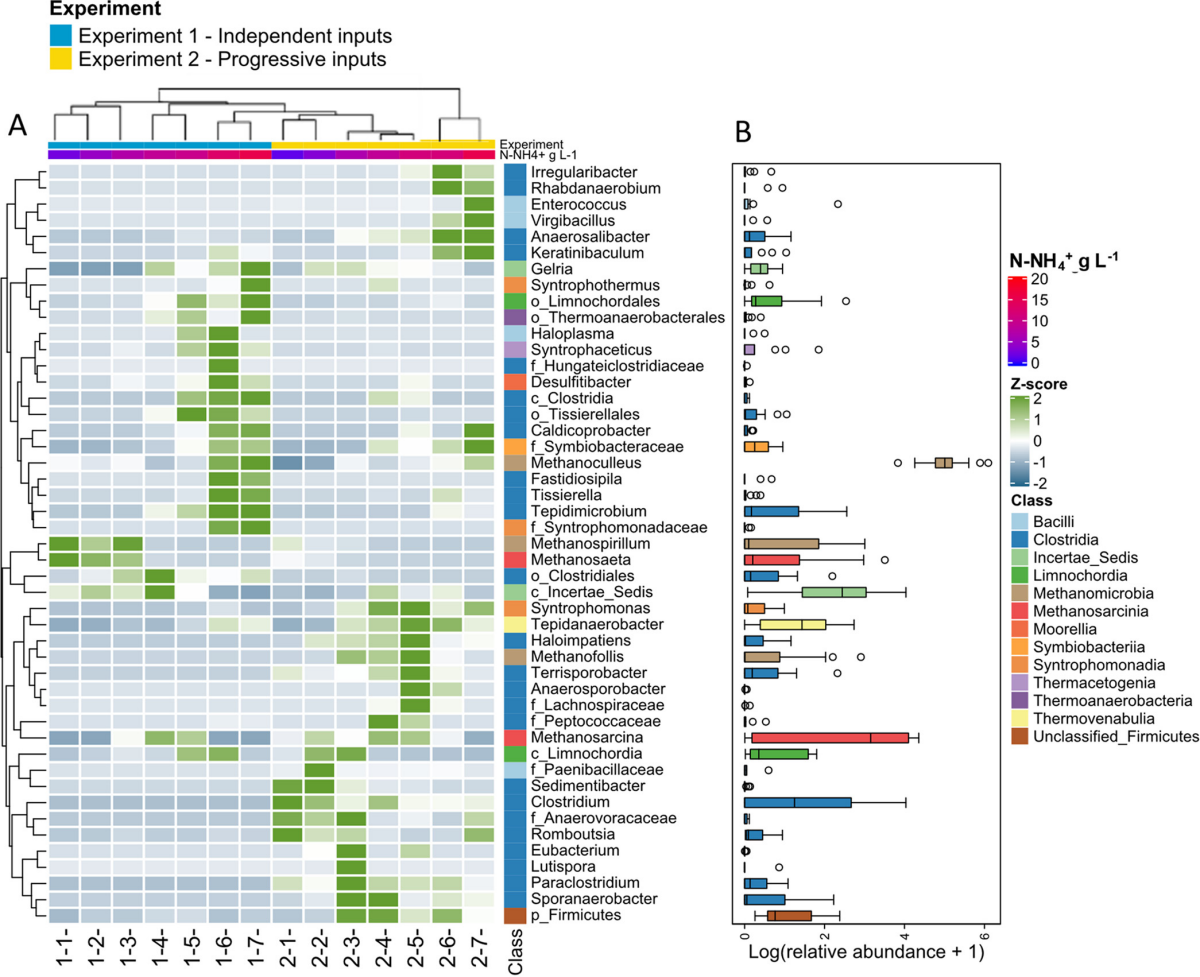
(A) Heatmap showing the normalized abundance (z-score, by row) of differentially expressed transcripts belonging to *Halobacterota* and *Firmicutes* phyla. The differentially expressed transcripts were selected by DESeq2 (adjusted *P* value of <0.05) and then aggregated at the genus level (or at the lowest available taxonomic level for unclassified genera, as identified by f_, family; o_, order; c_, class; p_, phylum). Each column represents the mean of biological duplicates. Samples and differentially expressed genera are arranged by hierarchical clustering based on their differential abundance patterns using Bray-Curtis distance. The vertical color code on the right side represents the taxonomic affiliation at the class level. The horizontal-colored bars at the top represent the ammonium concentration (from 1.7 to 15.3 g N-NH_4_^+^ L^1^) and the ammonium input modality (experiment1 and experiment 2). (B) Boxplot representing the distribution of the log-transformed relative abundances of each differentially expressed genus in the different samples.

To disentangle the effect of ammonium inputs (both quantitatively in terms of N-NH_4_^+^ concentrations and qualitatively in terms of shock modality) on individual members of the active communities, we focused on the two main phyla (*Halobacterota* and *Firmicutes*, representing jointly 76.4% ± 11.3% of the active community in both experiments) and identified the significantly differentially expressed taxa (DESeq2, *P*< 0.05) between the different conditions indicated in Table S5. The z-score pattern of the 47 most discriminant taxa clearly separated experiment 1 samples from experiment 2 samples, highlighting the preponderant effect of the N-input modality (Fig. 6). Within a given input modality, the 47 most discriminant taxa showed a clear succession following the NH_4_^+^ concentration gradient, as follows.

**Independent NH_4_^+^ input modality.** Other than unclassified *Firmicutes*, the overabundant active methanogens at low to medium NH_4_^+^ concentration (levels 1 to 3, up to 7.2 g N-NH_4_^+^ liter^−1^) belonged mainly to *Methanospirillum* and *Methanosaeta* (5.9% ± 1.5% and 7.7% ± 2.4% of the active community, respectively) (Fig. S5), which decreased down to <0.6% of the active community when N concentration increased to levels 3 to 4 (7.2 to 8.9 g N-NH_4_^+^ liter^−1^). At intermediary NH_4_^+^ concentrations (8.9 to 9.8 g N-NH_4_^+^ liter^−1^, levels 4 to 5), *Methanosarcina* was significantly overabundant, representing 17.8% ± 2.3% of the active community, and *Methanomassiliicoccus* reached 14.9% of the active community in one condition. The number of overabundant active genera remarkably increased at the highest NH_4_^+^ concentrations (12.8 to 15.3 g N-NH_4_^+^ liter^−1^, levels 6 to 7), including especially *Methanoculleus* (62.9 ± 6.4% of the active community) and several *Clostridia* members, such as *Tepidimicrobium* (up to 4.9% ± 0.1% of the active community). Other enriched taxa at the highest NH_4_^+^ concentrations belonged to *Tepidanaerobacter* (up to 3.1% of the active community), uncultured MBA03 group from the *Limnochordia* class (up to 2.3% of the active community), *Gelria* (around 1% of the active community), and other rare genera (0.1 to 0.6% of the active community), such as *Fastidiosipila*, *Tissierella*, *Caldicoprobacter*, and *Haloplasma*. Many active syntrophic taxa were also enriched at the highest NH_4_^+^ concentrations, belonging to *Syntrophaceticus* (up to 2.6% of the active community) and, to a lower extent, *Syntrophothermus*, unclassified *Syntrophomonadaceae*, and *Symbiobacteraceae* (0.1 to 0.7% of the active community).

**Successive NH_4_^+^ input modality.** We observed a very dynamic succession of differentially active fermentary *Firmicutes* members, characterizing short ranges of NH_4_^+^ concentrations. The overabundant active genera at low NH_4_^+^ concentrations (1.7 to 3.5 g N-NH_4_^+^ liter^−1^, levels 1 to 2) belonged mainly to *Clostridium* (13.0% ± 3.4% of the active community) and other minor (<1%) *Clostridia* members, such as *Sedimentibacter*, *Romboutsia*, and unclassified *Paenibacillaceae*. At intermediate NH_4_^+^ concentrations (6.7 to 8.8 g N-NH_4_^+^ liter^−1^, levels 3 to 4), other active *Clostridia* members appeared to be overabundant, such as *Sporanaerobacter* (3.7% ± 0.1% of the active community), *Paraclostridium*, and *Lutispora* (both around 1% of the active community). At intermediate to high NH_4_^+^ concentrations (8.8 to 10.4 N-NH_4_^+^ liter^−1^, levels 4 to 5), the overabundant active genera were affiliated with *Tepidanaerobacter* (3.7 to 5.7% of the active community), *Syntrophomonas* (around 1% of the active community), and *Clostridia* representatives, such as *Clostridium* (4.7 to 10.1% of the active community), *Terrisporobacter* (4.0% of the active community), *Haloimpatiens* (around 1%), and unclassified *Peptococcaceae* (<1% of the active community). The overabundant methanogens were affiliated with *Methanosarcina* (14.7% ± 3.2% of the active community) and *Methanofollis* (3.1 to 6.5% of the active community) over a wide range of low to medium NH_4_^+^ concentrations (levels 2 to 5, 3.5 to 10.4 g N-NH_4_^+^ liter^−1^). *Methanomassiliicoccus* never exceeded 2.2% of the active community (at 11.3 g N-NH_4_^+^ liter^−1^). At the highest NH_4_^+^ concentrations (levels 6 to 7, 11.3 to 15.1 g N-NH_4_^+^ liter^−1^), *Methanoculleus* became the overabundant active methanogen (41.7% ± 6.1% of the active community), together with *Enterococcus* (up to 4.1% of the active community) and other minor bacterial members (each representing 0.2 to 1.2% of the active community), such as *Virgibacillus*, unclassified *Symbiobacteraceae*, and other *Clostridia* members (*Keratinibaculum*, *Anaerosalibacter*, *Irregularibacter*, *Rhabdanaerobium*). As in experiment 1, the number of overabundant genera was higher at the highest N-NH_4_^+^ concentrations.

We also highlighted a clear succession at the ASV level within the dominant *Methanoculleus* genus along the ammonium gradient, suggesting that different ASVs were specific to different ecological niches defined by ammonium concentrations and exposure mode. This ASV succession was different according to the N-input modality, which was previously hindered at the genus level. For example, in experiment 1, increasing ammonium concentrations were associated with a progressive emergence of ASV_3 and ASV_24, both 100% similar to Methanoculleus bourgensis (BLAST, type strain HE964772 [27]). On the contrary, in experiment 2, the high ammonium concentrations favored ASV_13 and ASV_32, which had, respectively, 100% and 99.2% similarity to Methanoculleus receptaculi (NR_043961 [28]) and Methanoculleus chikugoensis (KP702949 [29]).

### Co-occurrence network.

**FIG 7:**
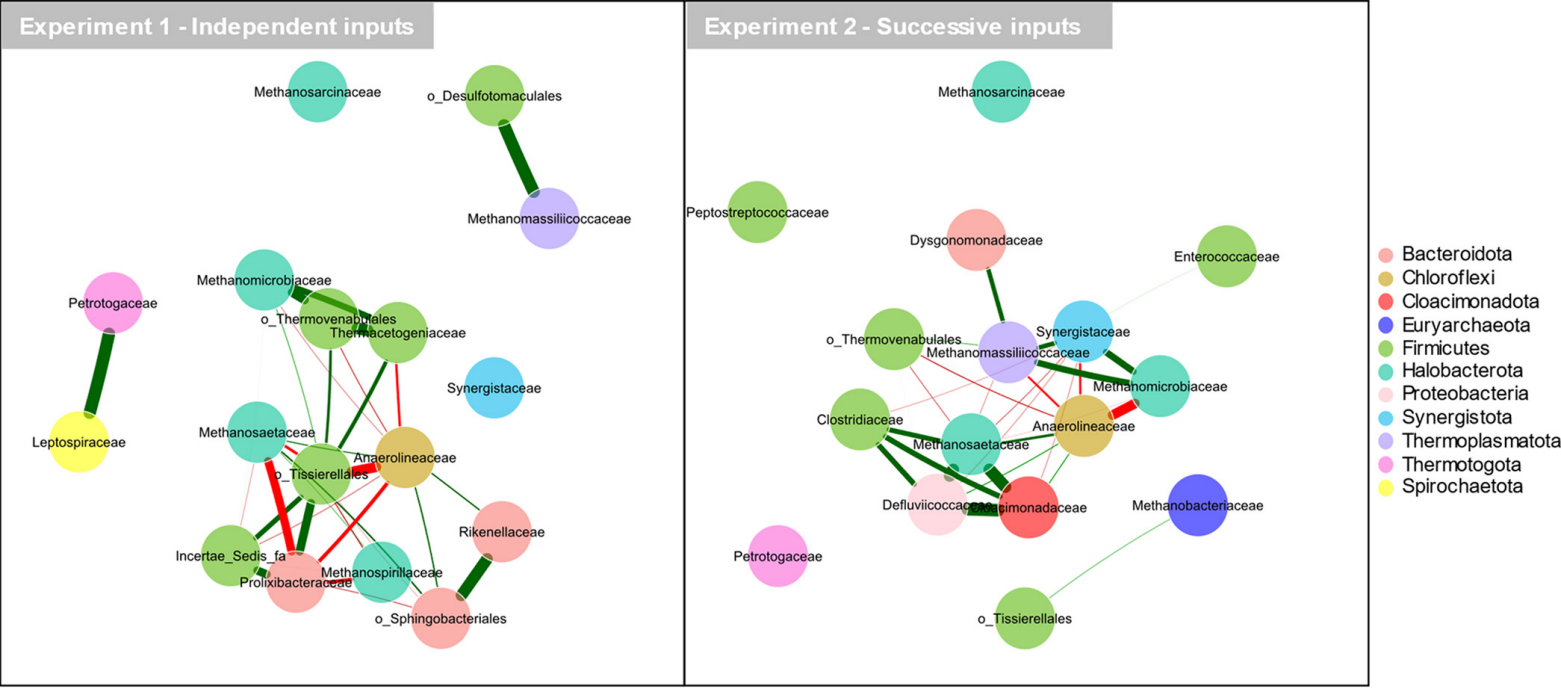
Co-occurrence networks of the 50 most abundant ASV aggregated at the family level, for (A) experiment 1 (independent inputs) and (B) experiment 2 (successive inputs), based on Pearson correlations. The thickness of the lines is proportional to the Pearson correlation coefficient *R*. Only correlations with |*R*| of >0.8 and *P* value of <0.05 are shown. Positive and negative correlations are shown by red and green lines, respectively. The nodes are colored according to the taxonomic affiliation at the phylum level.

For independent NH_4_^+^ inputs (Fig. 7A), the largest subnetwork showed two clusters of strongly connected families. In one cluster, *Methanosaetaceae* were positively associated with *Anaerolineaceae*, *Sphingobacteriales*, and *Methanospirillaceae*. The other cluster included, notably, *Methanomicrobiaceae* (*Methanoculleus*, *Methanofollis*) and *Firmicutes* (*Thermacetogeniaceae*, *Thermovenabulales*, *Tissierellales*) and was further connected with *Prolixibacteriaceae* and *Clostridia*. Both clusters showed strong negative correlations between each other. The subnetwork associating *Methanomassiliicoccaceae* and *Desulfotomaculales* was independent from the previous ones.

For successive NH_4_^+^ inputs (Fig. 7B), the main subnetwork was also divided in two clusters. In one cluster, *Methanosaetaceae* were positively associated with *Clostridiaceae*, *Cloacimonadaceae*, *Defluviicoccaceae*, and *Anaerolineaceae*. Most families from this cluster showed mutual exclusion with a second cluster, composed of *Methanomicrobiaceae* and *Methanomassilicoccaceae*, as well as *Synergistetaceae*, *Dysgonomonadaceae*, and *Thermovenabulales*. Contrary to the first experiment, *Methanomassiliicoccaceae* were not excluded from the main network, whereas an additional methanogenic family (*Methanobacteriaceae*) appeared positively associated with *Tissierellales*, separated from the main cluster.

Globally, both networks showed the same mutual exclusion between acetoclastic-centered (*Methanosaetaceae*-driven) and hydrogenotrophic-centered (*Methanomicrobiaceae*-driven) subnetworks. *Methanosaetaceae* played a key role in terms of connection numbers. In both experiments, *Anaerolineaceae* appeared as a keystone family, establishing the highest number of connections and acting as a pivotal point between the mutually excluding groups. However, the other bacterial families interacting with the methanogens diverged between both experiments. In particular, *Tissierellales*, *Prolixibacteriaceae*, and *Sphingobacteriales* had a key “hub” role in experiment 1 (5 to 8 connections), while they were marginal or absent in the experiment 2 network. Conversely, *Synergistaceae* and *Cloacimonadaceae* showed a key hub role in experiment 2 but were absent from experiment 1 network.

## DISCUSSION

### Effect of ammonia concentration and its supply modality on BMP performances.

At the lowest ammonium concentration, we reported BMP efficiency globally comparable with the “undisturbed” literature, even if efficiency indicators depend on substrates and inoculum (30, 31). Our MPR IC_50_ values (2.19 to 3.30 g N-NH_4_^+^ liter^−1^) were very close to the median value (3.9 ± 2.7 g N-NH_4_^+^ liter^−1^) computed from the recent survey of Capson-Tojo et al. (10). Inhibition magnitude depends on pH, temperature, inoculum, acclimation period, reactor configuration, antagonistic effect of other ions, and substrates (10, 32).

The lower lag phase duration in the successive experiment likely reflected the better adaptation of the microbial community to gradually increasing N concentrations. Microbial communities can adapt to successive perturbations of increasing intensity, thereby improving the response to future strong disturbances (33, 34). However, the other process indicators (MPR, sCOD removal efficiency) were inhibited more strongly by the successive N-input modality, revealing a detrimental cumulative effect of the successive ammonium shocks on these functional outcomes, than by direct exposure. The adaptive versus cumulative effect of multiple disturbances on ecosystem function is highly dependent on the type of perturbations and the targeted functional outcome (35). The system imbalance (indicated by VFA accumulation) was strongly worsened when the community was exposed to successively increasing intensity ammonium shocks compared to that when the application of a single shock of a given intensity, suggesting an additive effect. One explanation might be that, in experiment 2, once the system had shifted toward an inhibited/inefficient community under the effect of a given disturbing N concentration, it could not go back to a stable state to cope with the following N level.

### Effect of ammonia concentration on the complex prokaryotic communities.

**(i) Enriched taxa at low to middle ammonium concentrations (levels 1 to 3: 1.7 to 7.2 g N-NH_4_^+^ liter^−1^).** *Methanosaeta* (and to a lower extent *Methanospirillum*) were the overabundant active methanogens at the lowest N levels, especially in experiment 1. The advantage of *Methanosaeta*, obligate acetoclastic methanogen, at low acetate and ammonia concentrations has been observed frequently (e.g., references 36 and 37) and can be explained by its higher substrate affinity (38). Here, *Methanosaeta* and *Methanospirillum* collapsed to almost undetectable levels when NH_4_^+^ concentration increased, similarly to previous findings in continuous reactors at 3.9 g liter^−1^ (39). The sensitivity of *Methanosaeta* to ammonia has also been shown in pure culture (40). The higher acetate concentration in experiment 2 can explain lower *Methanosaeta* abundance. The hydrogenotrophic *Methanospirillum* is also usually restricted to low ammonia conditions (41) and was favored at the expense of *Methanosaeta* in acidified AD (42).

In both experiments, *Anaerolineaceae* occupied a key hub role in the co-occurrence networks, correlated with the acetoclastic methanogens at low N. This family comprises alkane degraders, providers of formate, acetate, H_2_, and CO_2_, previously reported as codominant with *Methanosaeta* in reactors with different substrates (43, 44). In experiment 2, the diversity of overabundant bacteria at low to intermediate N-NH_4_^+^ levels was higher than that in experiment 1, with a clear succession of differentiated taxa according to the NH_4_^+^ level. Most of these taxa were providers of methane precursors (e.g., hydrolyzing and H_2_-producing *Paenibacillaceae* [45] and SCFA-producing *Romboutsia* [46] and *Sedimentibacter* [47, 48]), with a dominance of *Clostridium*, known as one of the most efficient H_2_ producers, especially through the acidogenic pathway leading to acetate/H_2_ coproduction (45).

**(ii) Enriched taxa at intermediate ammonium concentrations (8.8 to 10.4 g N-NH_4_^+^ liter^−1^).** At the intermediate NH_4_^+^ level, *Methanosarcina* was overabundant in both experiments. *Methanosarcina* is the most substrate-versatile methanogen. It has higher growth rates at high acetate concentration and is highly robust toward different process impairments, such as ammonium and acetate concentrations up to 7 g liter^−1^ and 15 g COD liter^−1^ (49), with some species resisting extreme ammonium concentration up to 50 g liter^−1^ (50). *Methanosarcina* is often dominant in disturbed AD processes with high VFA load (21, 51, 52). The resistance of *Methanosarcina* might be due to its capacity to form multicellular aggregates, limiting the diffusion of toxic compounds to the cells. However, higher acetate and N-NH_4_^+^ concentrations hinder the quorum sensing system of *Methanosarcina*, leading to cell clusters disintegration (53, 54). This might explain that its overabundance was transitory over the N gradient in the present study. This genus can shift from acetoclastic to hydrogenotrophic methanogenesis when acid load changes (55). However, whether the same shift occurs under ammonium stress is unclear.

The range of ammonium concentration in which the H_2_-dependent methylotrophic *Methanomassiliicoccaceae* (56) emerged depended on the ammonium supply modality. In experiment 1, *Methanomassiliicoccaceae* cooccurred with *Desulfotomaculales*, non-sulfate-reducing syntrophic bacteria frequently found in methanogenic environments (57). In experiment 2, *Methanomassiliicoccus* seemed to be more resistant to higher ammonium concentrations, in association with synergists. *Synergistacea* are able to degrade monocarboxylic and long-chain fatty acids (LCFA; butyrate, isoheptanoate, oleate) to produce H_2_, acetate, and CO_2_ (46) and are involved in amino acid turnover (58). Their possible syntrophic association with hydrogenotrophic methanogens has been reported previously (59).

In addition to methanogens, intermediate NH_4_^+^ conditions were characterized by several overabundant bacteria acting as substrate providers for the methanogens through two main ways: VFA producers (all in the *Clostridia* class) or syntrophic VFA degraders producing H_2_ and CO_2_.

First, the overabundant acetate producers at intermediate NH_4_^+^ range were affiliated mainly with *Sporanaerobacter* (60) and *Terrisporobacter* (46) in experiment 2. Some other fermenting SCFA producers also emerged at intermediary NH_4_^+^ concentrations, albeit at lower abundance, such as *Lutispora* (61) and *Haloimpatiens* (62). Interestingly, most of these VFA producers are able to degrade proteins and/or amino acids, which might explain their capacity to tolerate elevated ammonia concentrations. For example, increasing ammonia concentration favored the presence of *Sporanaerobacter* in swine manure AD up to 7 g N-NH_4_ liter^−1^ (63). However, their overabundance is transitory, since these genera are usually inhibited at the highest TAN, as observed for *Lutispora* in AD of wastewater treatment plant (WWTP) sludge at 7.8 g TAN liter^−1^ (64).

Second, the most enriched syntrophic VFA oxidizer at intermediary TAN was *Tepidanaerobacter* in experiment 2. *Tepidanaerobacter* strains syntrophically oxidize acetate (65) or alcohols and lactate (66) to produce H_2_/CO_2_ in coculture with a hydrogenotroph such as *Methanoculleus*. They are also known for their ammonia tolerance, being isolated from sludge at 6.4 to 7 g N-NH_4_^+^ liter^−1^ (65). In full-scale thermophilic AD treating food wastewater, the abundance of the dominant *Tepidanaerobacter* correlated with increasing ammonia concentration up to 4.3 g N-NH_4_^+^ liter^−1^ (67). Interestingly, despite the tolerance of pure *Tepidanaerobacter* growth up to 7 g N-NH_4_^+^ liter^−1^, Wang et al. evidenced that their syntrophic cocultivation with *Methanoculleus* and *Methanobacterium* was more strongly inhibited, with undetectable methane production above 3 g N-NH_4_^+^ liter^−1^ (68). When methanogenesis is inhibited, the absence of syntrophic partner leads *Tepidanaerobacter* to shift to acetate production (69), which could be the case here. In addition, other syntrophic SCFA- (especially butyrate) and LCFA-oxidizing bacteria affiliated with *Syntrophomonas* (70, 71) were overabundant at the same NH_4_^+^ range in experiment 2, albeit in lower proportions. *Syntrophomonas* were abundant and tolerant to medium to high NH_4_^+^ concentration in several continuous reactors (up to 6.5 g TAN/liter [39, 72]) and batch assays (up to 25 g liter^−1^ [50]), but other studies reported their inhibition at TAN of ≥6 g liter^−1^ (22).

**(iii) Enriched taxa at highest ammonium concentrations (from 11.3 to 15.3 g N-NH_4_^+^ liter^−1^).** Independently of the N-input modality, hydrogenotrophic *Methanoculleus* strongly dominated the active community at all N concentrations, being particularly enriched at the highest TAN levels. As a fast-growing methanogen, active at high ammonia levels, Methanoculleus bourgensis (27) was successfully used in bioaugmentation of continuous AD processes to enhance methane production at high ammonia levels (5 to 11 g N-NH_4_^+^ liter^−1^) (73, 74). *Methanoculleus* was dominant in many anaerobic digesters operated at high TAN (72) or acetate (75) levels. As evidenced by SIP, *Methanoculleus* was the most active methane producer, in association with SAO bacteria, in swine manure incubations characterized by high acetate and ammonia concentrations (76).

Regarding bacteria, the highest NH_4_^+^ conditions corresponded to the highest diversity of overabundant taxa. Among them, *Tepidimicrobium* enrichment in experiment 1 was consistent with previous studies reporting its prevalence (also in association with *Methanoculleus*) in continuous AD of nitrogen-rich substrates up to 6.5 g TAN liter^−1^ (67, 77) and in batch assays at extreme ammonia concentration above 25 g liter^−1^ (50). However, depending on the operating conditions, *Tepidimicrobium* can also be inhibited at high TAN, as observed in high solid digester at 5 to 6 g N-NH_4_^+^ liter^−1^ (52). *Tepidimicrobium* is a protein degrader that can fuel methanogens through VFA production (67). Some *Tepidimicrobium* species also have cellulolytic and xylanolytic activities (78).

The main SAO overexpressed at the highest ammonium level was affiliated with *Syntrophaceticus*. Known for its ammonia tolerance, *Syntrophaceticus* has been isolated from AD operating at 6.4 g N-NH_4_^+^ liter^−1^ (79) and detected in ammonia-stripping AD (77). The amino acid degrader *Gelria*, known as syntrophic glutamate oxidizer (80), was also overabundant at high TAN in experiment 1, albeit in lower proportion. Several authors reported the correlation of *Gelria* abundance with ammonium concentration up to 5.4 to 7.8 g N-NH_4_^+^ liter^−1^ in AD (64, 81). Eventually, the importance of the *Clostridia* group MBA03 (*Limnochordia*) in experiment 1 at the highest TAN has to be highlighted. This group, abundant in AD (e.g., reference 82), is tolerant to medium to high NH_4_^+^ levels (83, 84). Members of group MBA03 are suggested to be electroactive (85) and potentially involved in SAO based on SIP analysis (86). Their co-occurrence with *Methanoculleus* could thus be interpreted as direct interspecies electron transfer (DIET)-syntrophy (83).

### Effect of NH_4_^+^ input modality on microbial succession.

Despite the similar patterns of methanogens succession, the different N-input modalities selected for totally distinct bacterial partners, mainly within acetate producers and syntrophic SCFA oxidizers. The direct exposure modality (experiment 1) was characterized by a progressive enrichment of VFA producers (mainly *Tepidimicrobium*) and syntrophic VFA oxidizers (mainly *Syntrophaceticus*, *Gelria*, and *Clostridia* group MBA03) with increasing NH_4_^+^ concentration. Their relative abundance constantly increased with NH_4_^+^ concentration. This microbial pattern could be related to a very limited VFA accumulation and better performance in terms of COD removal efficiency and MPR at high ammonium concentrations.

In contrast, the successive exposure modality (experiment 2) was characterized by a more dynamic succession of VFA producers (mainly *Clostridium*, *Sporanaerobacter*, and *Terrisporobacter*) and syntrophic VFA oxidizers (mainly *Tepidanaerobacter* and *Syntrophomonas*), each one characteristic of a narrow NH_4_^+^ niche. This microbial pattern could be related to a decrease of alpha diversity and higher archaeal expression level, as well as to strong VFA accumulation and lower performance in terms of COD removal efficiency and MPR at high ammonium concentrations. While the community changes were progressive in experiment 1, each successive increase of ammonium concentration was accompanied by drastic changes of overabundant bacterial partners in experiment 2.

The overabundance of syntrophic bacteria at high N-NH_4_^+^ was noticeable. However, very few of them were common to both modalities of NH_4_^+^ addition. *Tepidanaerobacter* and *Gelria* were overabundant in both experiments but at different NH_4_^+^ conditions. After successive NH_4_^+^ supplies at extreme concentrations, syntrophic acetate oxidation and hydrogenotrophic methanogenesis seemed uncoupled. There is no consensus to date on the rate-limiting organisms within syntrophic consortia, with contradictory results between hydrogenotrophic methanogens (21) and SAOB (68) identified as, respectively, the rate-limiting groups. The ammonium tolerance of SAOB such as Syntrophaceticus schinkii and Tepidanaerobacter acetatoxydans could be due to the absence of genes for ammonium uptake systems (79). Also, their presence is particularly consistent with the fact that they can outcompete the other acetate-utilizing groups only under higher acetate concentrations (87). From a genome-centric approach, Yan et al. (88) showed that the variable capabilities of different microbiomes to tolerate ammonia seemed to be connected with (i) the homeostatic system (i.e., regulation of cation uptake, osmoprotectant synthesis, pH maintenance) and (ii) the ability to provide the extra energy required for homeostasis regulation. In that sense, the shift to hydrogenotrophic methanogenesis that we observed can be interpreted as a more exergonic energy supply in response to ammonium stress.

Few studies evaluated the effect of ammonium supply modality on process performance and community structure. From radioisotopic analysis, Fotidis et al. (21) observed that the direction of the methanogenic pathway shift in response to ammonia increase (from 1 to 7 g N-NH_4_^+^ liter^−1^) depended on the previous sludge acclimation to N exposure. Namely, under mesophilic conditions, the stepwise acclimation process led to the maintenance of *Methanosarcinaceae*-mediated acetoclastic pathway, while the direct exposure of nonacclimated sludge led to a shift to SAO-coupled hydrogenotrophic methanogenesis (mediated by *Methanosarcinaceae*, *Methanomicrobiales*, and *Methanococcales* at the highest N-NH_4_^+^ concentration) (21). In another study of Zhang et al., direct TAN exposure was more conducive to the rapid succession of syntrophic acetogenic bacteria and their adaptation to the new environment than stepwise TAN increase (22). In contrast with these two studies, we observed that stepwise ammonia increase led to microbial shifts stronger and faster than those of direct N inputs, with stronger efficiency loss. This original result can be at least partly explained by distinct operational conditions. Indeed, the previous batch studies used differently adapted inocula (e.g., from full-scale animal manure digester) and different substrates at a lower COD level (e.g., synthetic food waste at 8.8 g COD liter^−1^), and above all, they considered a lower range of ammonium concentration (up to 7.5 g N-NH_4_^+^ liter^−1^ only). Altogether, our results highlighted the importance of considering these specific operating parameters in order to evaluate the ammonium effect on AD and gave some important insights on differential microbial community behavior facing ammonia increase.

### Conclusion.

Globally, independent input modality led to higher MPR and sCOD removal efficiency and lower VFA accumulation than successive input experiment. In contrast, progressive input experiment presented lower latency time and potential faster adaptation to perturbation. Independently of the N-input modality, the acetoclastic *Methanosaeta* had the lowest resistance to ammonium, the mixotrophic *Methanosarcina* culminated at intermediate ammonium concentrations, and the hydrogenotrophic *Methanoculleus* was the most resistant to highest ammonium levels, together with enriched syntrophic SCFA oxidizers. These results are in accordance with those of previous studies (10, 22, 89). Additionally, we highlighted the effect of ammonium concentration and input strategy on methanogenic structure at a fine phylogenetic resolution, resulting in different ASV selection within the *Methanoculleus* genus. Ammonium concentration and exposure mode drove niche differentiation at the genus and ASV levels. Compared to that of direct additions, the successive input strategy led to higher dynamism in the succession of VFA producers and syntrophic VFA oxidizers, each characterizing a narrower ammonium niche.

These results open promising perspective for a better management of ammonium disturbance in AD based on interactions between active microbiome key players. These results should be further evaluated with different inocula, and in continuous reactors, to take into account the crossed effect of feeding strategy (e.g., hydraulic retention time [HRT], organic loading rate [OLR], potential washout of active members), inoculum history, and ammonium concentration on microbial community dynamics and interspecies competition.

## MATERIALS AND METHODS

### Inoculum.

Sludge inoculum was collected from a recirculation loop in a full-scale anaerobic digester fed with activated sludge from a municipal wastewater treatment plant (Veolia Environment) operated at 37°C, located in La Crau, France (83). Sludge inoculum total solid content was 40 g total solids (TS) liter^−1^, whereas liquid fraction contained about 500 mg sCOD liter^−1^ and 1.7 g N-NH_4_^+^ liter^−1^. Inoculation was carried out after sludge centrifugation (20 min, 12,000 × *g*, 4°C). The obtained pellet (at 80 g VS liter^−1^) amount to inoculate was adjusted so that the final sludge VS content in each batch assay was 17.5 g liter^−1^.

### Substrate.

A synthetic influent mimicking raw agro-industrial wastewater was used as soluble substrate. Its composition was adapted from the SYNTHES substrate proposed by Aiyuk et al. as described in Table S2 (90). The substrate initial pH was 7.1. The sCOD:N:P of the synthetic substrate was 30:3:1, representative of raw agro-industrial wastewater (90). The substrate was prepared as a stock solution at 40 g soluble COD liter^−1^ so that the final sCOD content in each batch assay was 17.5 g liter^−1^, and final acetate and ammonium concentrations were, respectively, 5.6 g liter^−1^ and 1.7 g N-NH_4_^+^ liter^−1^ at the basal level (level 1).

### Experimental setup for BMP batch assays.

Biomethane potential (BMP) was measured in batch assays in glass serum bottles (57.2 ml) with a working volume of 28.6 ml (50% vol/vol headspace ratio). Preliminary experiments have been carried out to optimize the initial substrate to inoculum ratio (sCOD/VS) in BMP in order to obtain a methanization efficiency close to the theoretical value (theoretical methane production of 350 Nml g^−1^ COD, 96% efficiency) under basal N-NH_4_^+^ concentration (i.e., close to that measured in La Crau’s digester: 1.7 g N-NH_4_^+^ liter^−1^). As a result, an initial sCOD/VS ratio equal to 1 (wt/wt) was applied for all the subsequent BMP experiments (Fig. S1). Experimental vials were hermetically sealed with blue rubber stoppers (2048-11800A septum, Bellco) and flushed with N_2_ for 10 min just after assembling. The vials were incubated at 37°C in the dark without agitation. At the time of sampling (i.e., time of maximal activity, at the end of the exponential phase, as indicated in Fig. S2), the vial content was centrifuged at 12,000 rpm for 20 min at 4°C, and the pellet was immediately frozen in liquid nitrogen and stored at −80°C for molecular biology analysis, whereas the supernatant was stored at −20°C for subsequent analysis of VFA and sCOD concentrations.

### Experimental strategies for nitrogen inputs.

The influence of ammonium concentration on BMP efficiency was evaluated by adding different amounts of NH_4_Cl from a stock solution prepared at 200 g liter^−1^. The volumes of added NH_4_Cl and water were adjusted so that the final concentration ranged from 1.7 ± 0.1 to 15.1 ± 0.1 g N-NH_4_^+^ liter^−1^, representing a maximal increase factor of 8.9. Two strategies for nitrogen additions were compared (Fig. 1).

In experiment 1, each ammonium input was independent from the others. In the control condition 1-1, ammonium concentration was maintained at the in-situ level without further increase (i.e., 1.7 ± 0.1 g N-NH_4_^+^ liter^−1^, as in the inoculum). In parallel, in the 6 other conditions (1-2 to 1-7), independent ammonium inputs were applied directly on the initial inoculum sludge at 6 unique target concentrations (ranging between 4.8 ± 0.1 and 15.3 ± 0.9 g N-NH_4_^+^ liter^−1^). In experiment 1, batch assays lasted from 56 to 132 days for each level, according to the time necessary to complete methane conversion under the different ammonium concentrations. For each level, 6 vial replicates were set up: three of them were sacrificed at the end of the exponential methane production (as indicated by the red arrows in Fig. S1) and used for microbial community analysis and soluble compounds determination (sCOD and VFA), while the three other replicates were maintained to determine the maximal amount of methane production, further used for yield calculation.

In experiment 2, progressively increasing ammonium concentrations (ranging from 1.7 ± 0.1 to 15.1 ± 0.3 g N-NH_4_^+^ liter^−1^) were applied successively on the initial sludge in a stepwise manner. For this, 24 replicated vials were prepared at basal NH_4_^+^ concentration (1.7 ± 0.1 g liter^−1^). For each NH_4_^+^ level, once the maximal methanogenic activity was reached, three replicates were sacrificed at the end of the exponential phase for microbial community analysis and soluble compounds (sCOD and VFA) measurements, while the other replicates were exposed to the next ammonium level, as follows. The vial content was centrifuged under N_2_ atmosphere (20 min, 12,000 × *g*, 4°C). After centrifugation, the spent medium (supernatant) was discarded in order to avoid accumulation of (potentially toxic) culture intermediates or byproducts. Each pellet was transferred to a new vial with fresh substrate containing ammonium at the subsequent concentration level (Fig. 1). Fresh substrate was used to avoid problems of nutrient or trace element depletion. We could verify that the centrifugation and transfer steps from *n* to *n* + 1 level did not affect the biomass activity, since methane production began as soon as day 0 of incubation at the *n* + 1 level, without latency phase (Fig. S1). This instantaneous recovery indicated that the biomass was still highly active. The transfer operation was repeated 6 times, until the last level of ammonia concentration (15.1 ± 0.3 g N-NH_4_^+^ liter^−1^). In experiment 2, batch assays lasted for 159 days in total, with each ammonium level being maintained during 10 to 42 days depending on the concentration. Experiment 1 evaluated the resistance capacity of inoculum sludge toward a single given adverse N-NH_4_^+^input, whereas experiment 2 was designed to analyze the capacity of the microbial community to adapt through progressively increasing ammonia concentrations.

### Analytical methods.

**(i) Total solids and volatile solids.** Total solids (TS) content (% wt/wt) and volatile solids (VS) content (% wt/wt) were determined on 20 ml of homogenized sample after drying for 24 h at 105°C (for TS) and subsequent igniting for 2 h at 550°C in a muffle furnace (for VS), according to Clesceri et al., 1998 (91). TS and VS were determined in triplicate using a precision balance.

**(ii) Monitoring of methane production.** Pressure in the headspace was measured daily using a differential manometer (GDH 200-13, PCE). Headspace gas samples (40 μl) were collected through the septum with a gastight glass syringe in order to keep the actual vial pressure (push-pull 100-V-R-GT, SGE). The amount of methane present in the headspace was determined using a gas chromatograph with flame ionization detector (GC-FID Clarus 500, Perkin Elmer) equipped with a packed column (Chromosorb GAW/DMCS 80/100, 1.5 m, 2.5% SE 30 coating, Restek), with injector, oven, and detector temperatures of, respectively, 180°C, 80°C, and 220°C, and helium carrier flow at 35 ml min^−1^. CH_4_ amounts were directly converted into micromoles thanks to a standard curve at atmospheric pressure (from 0.001% to 50% vol/vol) with pure methane (99.8% Aldrich). The GC-FID calibration was verified each day of analysis in order to avoid any possible deviations.

After each measurement, the headspace was reequilibrated to atmospheric pressure by degassing with a 25G needle inserted through the septum (Agani needle, Terumo). At each time *t*, the effectively produced amount of CH_4_ (Δ*n*_CH4_, mol) during the time period between two measurements (*t* and *t* − 1) was obtained by the following equation:

$$\Delta n_{CH_{4}}=n_{CH_{4}}\left(t\right)\;-\;n_{CH_{4}}\left(t\;-\;1\right)=\frac{[(P_{\mathrm{atm}}\;+\;P_{\mathrm{HS}}\left(t\right))x_{CH_{4}}\left(t\right)\;-\;P_{\mathrm{atm}}x_{CH_{4}}(t\;-\;1)]V_{\mathrm{HS}}}{\mathrm{RT}}$$

with *P*_atm_ and P_HS_(*t*), respectively, the atmospheric pressure and the measured pressure in the headspace at time *t* (Pa), *V*_HS_ the headspace volume (m^3^), *x*_CH4_(*t*) and *x*_CH4_(*t* − 1) the molar fraction of CH_4_ in the biogas given by gas chromatography at time *t* and *t* − 1, respectively (% vol/vol), *T* the incubation temperature (K), and *R* the ideal gas constant (m^3^ Pa K^−1 ^mol^−1^). The produced methane amount was cumulated along time and converted to volume using normal temperature and pressure conditions (273 K, 101,325 Pa). All CH_4_ volumes are thus expressed in Nml (i.e., normal temperature and pressure [NTP] conditions) and will be abbreviated in the following text and figures as ml.

Methane production rate (MPR) was determined as the maximal linear slope of the methane production kinetics along time, estimated from 4 to 6 successive measurements, and normalized by the initial VS content in each vial. The latency phase was calculated as the minimal time for methane production to increase by 20% from its previous value. Methane production yield was calculated as the ratio of maximal produced CH_4_ volume to the amount of fed sCOD.

**(iii) Quantification of volatile fatty acids (VFA).** Concentrations of acetate, propionate, and butyrate were determined in 20 μl of supernatant using liquid chromatography (HPLC Spectra System, Thermo, equipped with a Bio-Rad Aminex 487H column and a refractive index detector RID-6A Shimadzu), carried out at 45°C, with 2.5 mM sulfuric acid as mobile phase at a flow rate of 0.6 ml min^−1^. Standard solutions between 5 and 10 mM were elaborated for each pure compound and used for external calibration. The limit of quantitation was 0.5 mM for each VFA.

**(iv) Quantification of ammonium and sCOD.** The soluble COD was quantified in the supernatant with an integral multiparameter photometer (HI83399-02, Hanna Instrument) using Hanna COD reagents based on the dichromate method (HI93754B-25 and HI93754C-25, depending on the concentration range, up to 1,500 mg O_2_ liter^−1^ and 15,000 mg O_2_ liter^−1^, respectively). Total ammonium nitrogen was determined using Hanna kit (Hanna instrument, AMMONIA HR, Iso Method [HI93764B-25]).

### Prokaryotic community analysis.

**(i) DNA and RNA extraction.** Total DNA and RNA were simultaneously extracted from 0.5 g of sludge pellet (in duplicate) using RNeasy PowerSoil total RNA kit and RNeasy PowerSoil DNA elution kit (Qiagen) and eluted in 50 μl of molecular biology grade water. DNA contamination in RNA extracts was removed using the TURBO DNA-free kit (Ambion, Life Technologies) according to the manufacturer’s instructions, applying two successive DNase treatments (rigorous and classic cycles). The DNA and RNA quantification and absorbance ratios at 260/230 and 260/280 were measured by spectrophotometry (BioSpec-nano, Shimadzu). The absence of DNA contamination in RNA samples was verified by the absence of PCR amplification (as described below) on the 16S rRNA gene (1 ng of total RNA per reaction) before the reverse transcription step.

Reverse transcription was carried out on 1 ng of total RNA using SuperScript IV reverse transcriptase (Life Technologies) with a random primer (250 ng per reaction) according to the manufacturer’s instructions. Dilution of 1:10 of synthesized cDNA was used for subsequent quantitative PCR (qPCR).

As an indicator of protein synthesis potential, the 16S RNA-based community corresponds to the actively transcribing prokaryotes and will be referred to in the following text as “active community.”

**(ii) Bacteria and archaea quantitative PCR.** Absolute quantification of bacterial and archaeal 16S rRNA genes and transcripts was performed by qPCR using specific primers and the procedure described in Table S3. Each (real-time [RT])-qPCR (20 μl) contained 2 μl of DNA or cDNA (about 0.4 ng of template DNA), 0.5 μM concentration of each primer, and 10 μl of SsoAdvanced SYBR Green Supermix (Bio-Rad) and was performed in duplicate on CFX96 real-time system (Bio-Rad Laboratories, Hercules, CA, US) using Bio-Rad CFX Manager 3.1 software (184500; Bio-Rad CFX Manager). The specificity of the amplification products was verified by the melting curve (between 65°C to 95°C, increase of 0.5°C with a time step of 5 sec).

**(iii) 16S rRNA gene and transcript sequencing.** For ribosomal diversity analysis of both total (DNA-based) and active (cDNA-based) communities, the V4 region of the bacterial and archaeal 16S rRNA genes was amplified using the universal primer set 515F-806R (0.5 μM final) and Q5 Hot Start high-fidelity 2× master mix (M0494S, New England Biolabs) according to the protocol detailed in Table S3. The 16S amplicons were sequenced on MiSeq Illumina technology (paired end 2 × 250 bp) using MiSeq reagent kit v3 (Illumina- MS-102-3003) and PhiX Sequencing Control V3 (Illumina FC-110-3001). 16S-rRNA gene and transcript raw read sequences are deposited in public database GenBank (accession numbers KEXZ01000001 to KEXZ01004520).

### Bioinformatic analysis.

Raw sequences were resolved into amplicon sequence variants (ASVs) with the DADA2 pipeline (version 1.16) using default settings to correct sequence errors in the R software version 3.4.3 (92). This method does not impose the arbitrary dissimilarity threshold defining operational taxonomic units (OTUs) and enables to distinguish sequence variants differing by as little as one nucleotide. Sequences (without primers and adapters) were filtered and further trimmed (removing 15 nucleotides [nt] at both 5′ and 3′ ends and setting a minimum length of 150 bp), quality checked, and denoised (*learnError*). DADA2 denoising is based on error distribution differing between R1 and R2 reads. For quality check, the maximum number of “expected errors” allowed in a read was 1 for reverse and forward reads. In other terms, all the sequences in which there was at least one read with high probability of erroneous base assignment (>63%) were discarded. True sequence variants were inferred for each sample from the unique (dereplicated) sequences. Forward and reverse reads were merged (overlap of at least 12 bases, with identical bases in the overlap region). Chimeras were removed (*removeBimeraDenovo* function, with the consensus method).

One sample (cDNA sequences from one replicate of the 2 to 3 condition) presenting too low a number of reads (5,148) was removed (Table S4). After quality filtering, a total of 18,389,369 high-quality DNA- and RNA-based sequences were obtained (ranging from 60,229 to 166,737 sequences per sample), with an average length of 258 bp (Table S4; Fig. S3). The Good’s coverage index showed that the sequencing depths covered 98% of the microbial diversity and the rarefaction curves approached the saturation (Fig. S3). All sequences were distributed into 4,952 ASVs, with each sample containing between 149 and 1,222 ASVs.

Taxonomy was assigned to the output sequences through the Silva database (version 138). ASV count and taxonomy tables produced by the DADA2 pipeline were imported into *phyloseq* v1.30.0.

All statistical analyses were conducted in R 3.4.3, using mainly the *vegan* package 2.5.7 and *phyloseq* package 1.30.0. Alpha diversity was calculated by the observed richness and Shannon index. The effect of ammonium concentration and perturbation modality on observed richness and Shannon index was assessed by analysis of variance (ANOVA; *aov* function, *stats* package 3.6.1) and *post hoc* test and Spearman correlations (*cor* and *rcorr* functions from packages *stats* and *Hmisc*, after verifying the nonnormality of the data with the *shapiro.test* function).

Differences in community composition were explored by principal coordinate analysis (PCoA, *ordinate* function) computed from the Bray-Curtis dissimilarity index, after rarefaction of the ASV table (*rarefy_even_depth* function) to the depth of the less abundant sample (60,229 counts). The clustering significance on PCoA representation was tested by nonparametric permutational multivariate analysis of variance (PERMANOVA, 99 permutations) using Bray-Curtis distance matrix. Significant clustering was represented on the PCoA plot by ellipses built at 80% confidence interval level (*stat_ellipse* function). The contribution of different explanatory variables (i.e., targeted fraction of the community, NH_4_^+^ concentration, NH_4_^+^ input modality) on community structure was evaluated by a variation partitioning analysis (*varpart* function). To better identify the drivers of active microbial communities, PCoAs were also computed for each experiment separately, including only the active subset of each community. On these ordinations, physicochemical variables (ammonium, MPR, residual COD and VFA concentrations) were represented by arrows pointing to the direction of the increasing gradient (*envfit* function). The arrow length was proportional to the correlation coefficient between the variable and the ordination axis, while the arrow color represented the *P* value of the correlation (permutation test with 99 permutations). In addition, for each experiment separately, the 50 most abundant active ASVs belonging to 3 dominant archaeal phyla and 6 dominant bacterial phyla were aggregated at the family level, represented on each ordination, and their significant correlation was tested with the *envfit* function. The taxonomic composition of the community was represented by bar plots displaying the top 11 most abundant phyla (representing more than 75% of the total communities) using *microbiome* package 1.10.0 (*aggregate_top_taxa* function). All phyla with relative abundance lower than 1% of the total community were represented in the group “other.”

The package *DESeq2* (1.28.1) was used to identify the ASVs whose abundances changed significantly between different pairwise conditions (adjusted *P* < 0.05). For both experiments, the selected pairwise comparisons are described in Table S5 and enabled to compare (i) each NH_4_^+^ level with the previous one, (ii) each NH_4_^+^ level with the initial one, and (iii) each NH_4_^+^ level with the corresponding one in the other experiment. For this analysis, we chose to focus on the active (RNA-based) communities of two most abundant phyla of higher ecological interest in our process: *Halobacterota* for archaea (encompassing most methanogenic classes in the new Silva classification v138) and *Firmicutes* for bacteria. The 161 differentially abundant ASVs from these two phyla were then aggregated at the genus level or at the immediately upper available informative level, resulting in 47 differential taxa. Each taxon abundance was the sum of its ASV abundances. The normalized relative abundances of the 47 differential genera from *Halobacterota* and *Firmicutes* were visualized on a heatmap (*ComplexHeatmap* package) based on the z-score of each ASV (i.e., the distance from the mean abundance, expressed in number of standard deviations, by row). A box plot representing the relative abundance of each genus (expressed as log) was generated and included on the heatmap plot, by the *row_anno_boxplot* and *rowAnnotation* functions. The 47 selected genera and the 14 samples were arranged by hierarchical clustering on the heatmap plot, based on Bray-Curtis distance computed from the z-score pattern.

Co-occurrence networks were built using the 50 most abundant ASVs from the active communities of experiment 1 and experiment 2, separately, after agglomeration at the family level. The co-occurrence of microbial families was evaluated calculating the Pearson correlation using the function *cor* in the R package *stats*. Correlation networks were plotted using *qgraph* package 1.6.9., representing only significant correlations (*P* < 0.05) with an absolute Pearson coefficient higher than 0.8.

The impact of ammonium concentration and ammonium exposure mode on functional indicators was evaluated by the nonparametric Kruskal-Wallis (KW) test.

### Data availability.

The targeted locus sequences (TLS) have been deposited at NCBI GenBank under the BioProject number PRJNA752008, accession KEXZ00000000. The version described in this paper is the first version and consists of curated unique sequences KEXZ01000001 to KEXZ01004520.
