# Supplementary material for: Similar Methanogenic Shift but Divergent Syntrophic Partners in Anaerobic Digesters Exposed to Direct versus Successive Ammonium Additions
Source: Microbiol Spectr. 2021 Oct 6;9(2):e00805-21. doi: 10.1128/Spectrum.00805-21 (PMC8510171; doi:10.1128/Spectrum.00805-21)
Supplement: Supplemental file 1 — Supplemental material. Download SPECTRUM00805-21_Supp_1_seq1.docx, PDF file, 0.7 MB [file spectrum00805-21_supp_1_seq1.docx]

**Supplemental Material for:**

**“Similar methanogenic shift but divergent syntrophic partners in anaerobic digesters exposed to direct versus successive ammonium additions”**

Authors:

Julie Hardy ^1,2 *,^ Patricia Bonin^1^, Adele Lazuka^2^, Estelle Gonidec^2^ , Sophie Guasco^1^ ,Corinne Valette^1^ , Sébastien Lacroix^2^ , Léa Cabrol ^1,3 *^

1. *Aix Marseille University, Univ Toulon, CNRS, IRD, MIO, Marseille, France*
2. *Veolia, Scientific & Technological Expertise Department, Chemin de la Digue, F-78600 Maisons-Laffitte, France*
3. *Instituto de Ecologia y Biodiversidad (IEB) Facultad de Ciencias, Universidad de Chile Las Palmeras, 3425 Nunoa, Santiago, Chile*

*Corresponding author


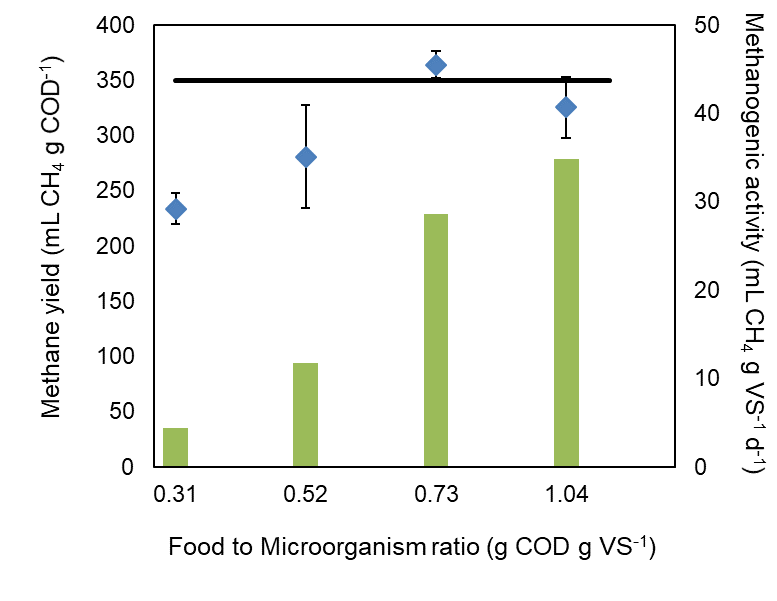


**Figure S1.** Optimisation of substrate to inoculum ratio (sCOD/VS, w/w) in batch assays in order to obtain a methanization efficiency close to the theoretical methane production of 350 NmL CH_4_ g^-1^ COD (black line) under basal N-NH_4_^+^ concentration (i.e. close to that measured in the inoculum: 1.7 g N-NH_4_^+^ L^-1^). Methane yield is represented as blue dots, methanogenic activity is represented as green bars.

**Figure S2.** Kinetics of methane production along time, in **(A)** Experiment 1 (independent inputs) and **(B)** Experiment 2 (successive inputs). In Experiment 1, red arrows represent the sampling time for microbial community analysis and soluble compounds quantification (VFA, COD). Mean and standard errors measured from on 6 and 6 to 24 replicates for Experiment 1 and 2 respectively, are represented.

**Figure S3:** Rarefaction curves for the 28 samples showing the detected diversity. The x axis represents the number of sequences, the y axis represents the number of ASV per sample.

**Figure S4:** Richness (observed OTU number) and Shannon diversity index of the total (DNA-based, ⚫) and active (RNA-based, **⭘**) communities, according to total ammonium nitrogen (N-NH_4_^+^) concentration in Experiment 1 (independent N inputs) and Experiment 2 (successive N inputs). Values from duplicate incubation vials are represented.

**Figure S5:** Taxonomic affiliation and relative abundances of the methanogenic Archaea from Halobacterota, Thermoplasmatota and Euryarcheaeota phyla, obtained from the 16S rRNA gene (DNA) and transcripts (RNA) sequences, represented at the genus level.

**Supplementary Table S1.** Literature survey showing examples of high ammonium concentrations in industrial wastewaters.

| **N-NH_4_^+^ concentration (mg L^-1^)** | **Wastewater origin** | **Reference** |
| --- | --- | --- |
| 800 | Mixed effluents from biological treatment (anaerobic digestion and oxygen stabilization) of the organic fraction of municipal solid waste | 1 |
| 1500 – 2000 | Fertilizer industry | 2 |
| 600 – 900 | Manufacture of ammonium nitrate and urea fertilizers | 3 |
| 40 | Municipal wastewater | 4 |
| 4200 | Supernatant from anaerobically digested swine wastewater effluent | 5 |
| 3820 | High strength animal wastewater | 6 |
| >6000 | Sulfidic caustic solutions from the oil-refining industry | 7 |
| 37 - 820 | Different types of industrial wastewater  (dumps leakage, opto-electronic, coal gasification, wool textile, dairy industries) | See references in 8 |
| 200-500  600-700  1200  2200 | Tannery  Sludge dewatering  Leachate  Landfill leachate | See references in 9 |
| 4000-6000 | Real wastewater from chemical and fermentation/distillation industries | 9 |
| 6000 | Urban landfill leachate | 10 |
| 2300  2740-4950  5000  2300 | Biologically treated blue crab processing effluent  Piggery wastewater  Raw manure digestate  Sludge liquor from municipal WWTP | See references in 11 |
| 6700 - 10200 | Coal gasification process | 12 |
| 300 - 3500 | Slaughterhouse wastewater | 13 |

Supplementary Table S2. Composition of the SYNTHES substrate (adapted from Aiyuk and Verstraete, 2004)

| **Name of the component** | **Molecular mass (g.mol^-1^)** | **Quantity (mg. L^-1^)** |
| --- | --- | --- |
| Na-acetate, 3H_2_O | 136.08 | 5 625 |
| Peptone |  | 750 |
| Dried yeast |  | 2 250 |
| Starch |  | 5 250 |
| Milk powder |  | 5 000 |
| MgHPO_4_, 3H_2_O | 174.33 | 500 |
| K_2_HPO_4_, 3H_2_O | 174.18 | 400 |
| Urea | 60.06 | 4 000 |
| NH4Cl | 53.49 | 1 700 |
| FeSO_4_, 7H_2_O | 198.81 | 625.0 |
| Cr(NO_3_)_3_, 9H_2_O | 400.15 | 937.5 |
| CuCl_2_, 2H_2_O | 170.48 | 625.0 |
| MnCl_2_, 4H_2_O | 169 | 119.7 |
| NiCl_2_, 6H_2_O | 282.85 | 282.6 |
| ZnCl_2_ | 136.29 | 312.5 |
| CaCl_2_ | 110.98 | 625.0 |
| Overall parameters | | |
| DCO_total_ |  | 40 000 |

Supplementary Table S3. PCR and qPCR primer sequences and concentrations (A) and amplification cycles (B). The procedure for standard curves validation is described (C).

A.

| Primer | Target gene | Sequence (5’-3’) | Reference | Fragment size (bp) | Final concentration (µM) |
| --- | --- | --- | --- | --- | --- |
| DGGE300F | 16S rRNA Bacteria (qPCR) | GCCTACGGGAGGCAGCAG | 14 | 172 | 0.25 |
| univ516 |  | GTDTTACCGCGGCKGCTGRCA | 15 |  |  |
| Arc 931F | 16S rRNA Archaea (qPCR) | AGGAATTGGCGGGGGAGCA | 16 | 169 | 0.25 |
| m1100R |  | BTGGGTCTCGCTCGTTRCC | 17 |  |  |
| 515F | 16S rRNA Archaea and Bacteria, V4 region (sequencing) | TCGTCGGCAGCGTCAGATGTGTATAAGAGACAGGTGYCAGCMGCCGCGGTAA | 18 | 292 | 0.5 |
| 806R |  | GTCTCGTGGGCTCGGAGATGTGTATAAGAGACAGGGACTACHVGGGTWTCTAAT |  |  |  |

B.

|  | **qPCR Bacteria** | | | **qPCR Archaea** | | | **Prokaryotes sequencing** | | |
| --- | --- | --- | --- | --- | --- | --- | --- | --- | --- |
| Initial denaturation  Denaturation  Hybridization  Elongation  Final elongation | 98°C  98°C  55°C  72°C   95°C | 2 min  5 s  10 s  12 s   10 s | 30 times | 98°C  98°C  62°C  72°C  95°C | 2 min  10 s  10 s  12 s   10 s | 35 times | 98°C   98°C  62°C  72°C  72°C | 30 s  5 s  30 s  60 s   3 min | 30 times |
| Melting curve | 65°C to 95°C 05°C s^-1^ | | | 65°C to 95°C 0.5°C s^-1^ | | | 65°C to 95°C 0.5°C s^-1^ | | |

C.

|  | **qPCR Bacteria** | **qPCR Archaea** |
| --- | --- | --- |
| Efficiency | 130% | 98.3% |
| R2 | 0.989 | 0.999 |
| Slope | 2.764 | 3.363 |
| Y-int | 35.141 | 43.360 |

Standard curves were generated over five orders of magnitude, i.e. from 10^3^ to 10^8^ copies of template, using a plasmid containing specific marker genes. Standards used for total Bacteria, and total Archaea quantification corresponded to pGEMT plasmids harbouring a Gammaproteobacterial (*Pseudomonas stutzeri*) or a MG-II Euryarchaeaotal SSU rRNA gene fragment (clone Arch508, accession number HE647171) respectively. The qPCR efficiency was calculated according to the equation *E* = [10(−1/slope)−1]. Possible inhibitory effects were checked by spiking samples with a range of known concentrations of the plasmid.

**Supplementary Table S4.** Summary of the number of forward (A) and reverse (B) reads obtained by 16S rRNA gene and transcript sequencing, selected after the different pretreatment steps of raw sequences through the Dada2 pipeline, and final percentage of cleaned sequences used for further analyses. “DNA” and “RNA” refer to, respectively, 16S rRNA gene and transcript.

A.

| **Sample** | **Number of sequences:** | | | | | **Percentage of Final retained sequences** |
| --- | --- | --- | --- | --- | --- | --- |
|  | **input** | **filtered** | **denoised** | **merged** | **nonchimeric** |  |
| 1-1A-DNA | 198055 | 92760 | 89834 | 82920 | 72394 | 36.55 |
| 1-1A-RNA | 238871 | 99259 | 97732 | 89263 | 77978 | 32.64 |
| 1-1C-DNA | 242721 | 114022 | 110905 | 102513 | 90610 | 37.33 |
| 1-1C-RNA | 414055 | 183095 | 180918 | 166824 | 144307 | 34.85 |
| 1-2A-DNA | 243766 | 111956 | 108507 | 98541 | 84072 | 34.49 |
| 1-2A-RNA | 249867 | 106121 | 104533 | 93891 | 81690 | 32.69 |
| 1-2C-DNA | 160055 | 94471 | 91262 | 84749 | 71884 | 44.91 |
| 1-2C-RNA | 319210 | 85675 | 84010 | 75335 | **60229** | 18.87 |
| 1-3A-DNA | 222284 | 128905 | 124908 | 115954 | 96591 | 43.45 |
| 1-3A-RNA | 273980 | 105990 | 105103 | 97714 | 80474 | 29.37 |
| 1-3C-DNA | 228298 | 127135 | 123240 | 114305 | 94980 | 41.60 |
| 1-3C-RNA | 299976 | 123799 | 122706 | 114933 | 103143 | 34.38 |
| 1-4A-DNA | 212943 | 123174 | 119039 | 109231 | 90871 | 42.67 |
| 1-4A-RNA | 370938 | 197669 | 195996 | 182515 | 160866 | 43.37 |
| 1-4C-DNA | 286062 | 149901 | 144799 | 131263 | 108929 | 38.08 |
| 1-4C-RNA | 466558 | 176707 | 175729 | 165390 | 149470 | 32.04 |
| 1-5A-DNA | 342928 | 182726 | 177201 | 161496 | 135479 | 39.51 |
| 1-5A-RNA | 295142 | 130282 | 129100 | 118443 | 104103 | 35.27 |
| 1-5C-DNA | 366589 | 193346 | 187673 | 171834 | 142847 | 38.97 |
| 1-5C-RNA | 328268 | 149999 | 148828 | 140559 | 127016 | 38.69 |
| 1-6A-DNA | 319989 | 149109 | 144433 | 131768 | 111154 | 34.74 |
| 1-6A-RNA | 405768 | 121675 | 121161 | 116897 | 105205 | 25.93 |
| 1-6C-DNA | 335521 | 194075 | 188798 | 173879 | 143403 | 42.74 |
| 1-6C-RNA | 401858 | 143729 | 142902 | 136909 | 130357 | 32.44 |
| 1-7A-DNA | 291088 | 161716 | 157740 | 147451 | 128093 | 44.00 |
| 1-7A-RNA | 377203 | 112950 | 112582 | 109952 | 108718 | 28.82 |
| 1-7C-DNA | 321915 | 171972 | 167000 | 154346 | 129707 | 40.29 |
| 1-7C-RNA | 377959 | 78193 | 77773 | 74924 | 71246 | 18.85 |

B.

| **Samples** | **Number of sequences:** | | | | | **Percentage of Final retained sequences** |
| --- | --- | --- | --- | --- | --- | --- |
|  | **imput** | **filtered** | **denoised** | **merged** | **nonchimeric** |  |
| 2-1A-DNA | 402565 | 206433 | 198041 | 181383 | 166737 | 41.42 |
| 2-1A-RNA | 330567 | 170114 | 165844 | 150273 | 133914 | 40.51 |
| 2-1C-DNA | 340371 | 167163 | 158976 | 142547 | 132864 | 39.04 |
| 2-1C-RNA | 319033 | 156384 | 151633 | 135519 | 120917 | 37.90 |
| 2-2A-DNA | 328852 | 173335 | 165536 | 148355 | 132552 | 40.31 |
| 2-2A-RNA | 338594 | 174664 | 171174 | 153408 | 130789 | 38.63 |
| 2-2C-DNA | 409469 | 207610 | 199257 | 179903 | 158007 | 38.59 |
| 2-2C-RNA | 334392 | 174944 | 170898 | 152800 | 131324 | 39.27 |
| 2-3A-DNA | 300232 | 134886 | 129607 | 114416 | 96597 | 32.17 |
| 2-3A-RNA | 225865 | 107419 | 105380 | 91290 | 76546 | 33.89 |
| 2-3C-DNA | 402565 | 206433 | 198041 | 181383 | 166737 | 41.42 |
| 2-3C-RNA | 121657 | 7164 | 6839 | 5561 | 5148 | 4.23 |
| 2-4A-DNA | 421993 | 236280 | 228484 | 207363 | 166100 | 39.36 |
| 2-4A-RNA | 397106 | 234306 | 227409 | 207975 | 161097 | 40.57 |
| 2-4C-DNA | 358728 | 160517 | 158772 | 141184 | 103092 | 28.74 |
| 2-4C-RNA | 419283 | 226680 | 219863 | 198414 | 150803 | 35.97 |
| 2-5A-DNA | 393660 | 197284 | 195062 | 171348 | 143824 | 36.53 |
| 2-5A-RNA | 438691 | 236769 | 232274 | 216352 | 170422 | 38.85 |
| 2-5C-DNA | 365622 | 208229 | 206231 | 185040 | 150166 | 41.07 |
| 2-5C-RNA | 431525 | 208905 | 204308 | 186581 | 151784 | 35.17 |
| 2-6A-DNA | 346313 | 169392 | 167879 | 153364 | 131425 | 37.95 |
| 2-6A-RNA | 437030 | 187952 | 184361 | 169157 | 148767 | 34.04 |
| 2-6C-DNA | 212638 | 103518 | 102504 | 94822 | 74372 | 34.97 |
| 2-6C-RNA | 370107 | 154107 | 150957 | 137594 | 117105 | 31.64 |
| 2-7A-DNA | 392467 | 195267 | 192902 | 171133 | 136195 | 34.70 |
| 2-7A-RNA | 503919 | 189205 | 185655 | 168363 | 142625 | 28.30 |
| 2-7C-DNA | 300475 | 123162 | 121471 | 108655 | 88982 | 29.61 |
| 2-7C-RNA | 351576 | 135689 | 133016 | 121408 | 102490 | 29.1515917 |

**Supplementary Table S5.** Design of pairwise comparison between samples for DESeq2 analysis and resulting number of selected ASV for each comparison. The objective was to compare (i) each N-NH_4_^+^ level with the previous one, (ii) each N-NH_4_^+^ level with the initial one, (iii) each N-NH_4_^+^ level with the corresponding one in the other Experiment. The abundance of the resulting 47 unique ASVs is plotted as heatmap in Figure 5.

**
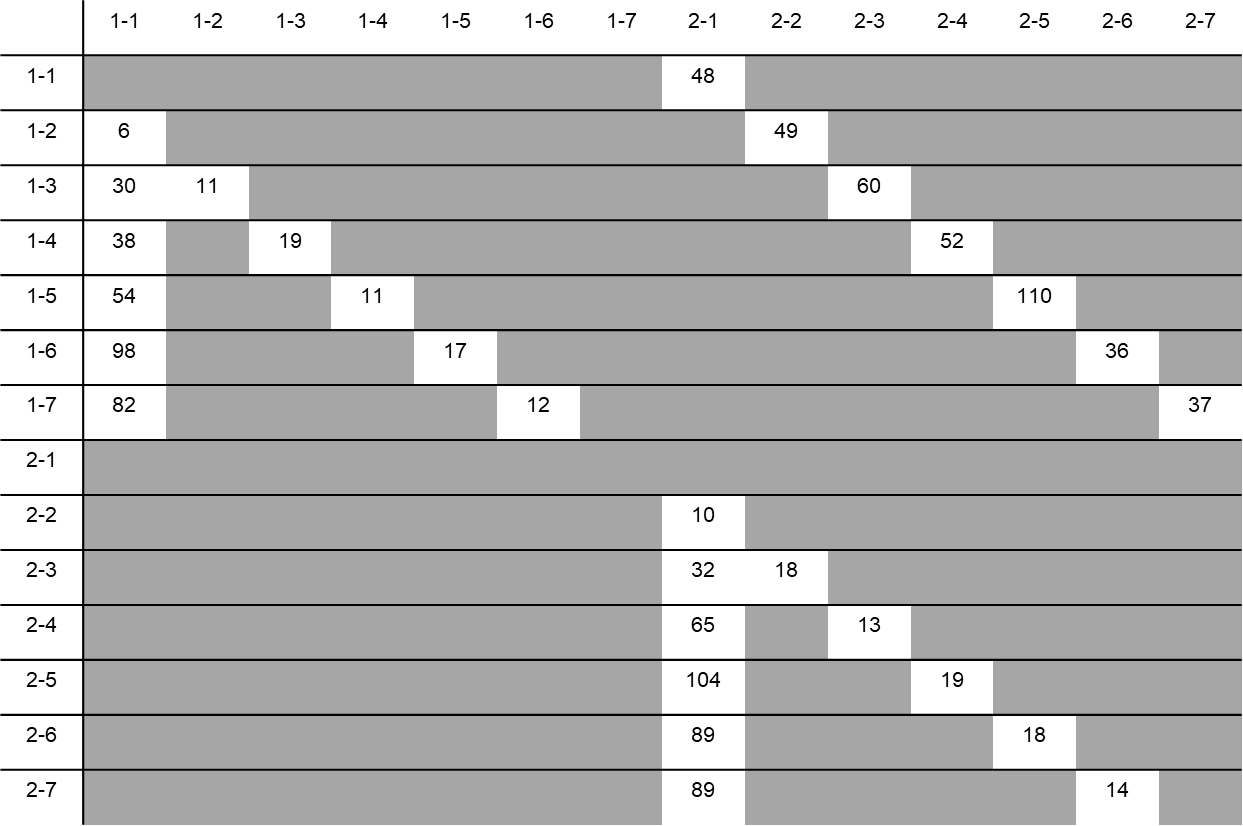
**

**References used in Supplemental Material:**

1. Seruga, P., Krzywonos, M., Pyżanowska, J., Urbanowska, A., Pawlak-Kruczek, H., & Niedźwiecki, Ł. (2019). Removal of ammonia from the municipal waste treatment effluents using natural minerals. *Molecules*, *24*(20), 3633.

2. Ahmed, A., Jyothi, N., & Ramesh, A. (2017). Improved ammonium removal from industrial wastewater through systematic adaptation of wild type Chlorella pyrenoidosa. *Water Science and Technology*, *75*(1), 182-188.

3. Hutton, W., & LaRocca, S. (1975). Biological Treatment of Concentrated Ammonia Wastewaters. *Water Pollution Control Federation*, 47(5), 989-997.

4. Malovanyy, A., Sakalova, H., Yatchyshyn, Y., Plaza, E., & Malovanyy, M. (2013). Concentration of ammonium from municipal wastewater using ion exchange process. *Desalination*, *329*, 93-102.

5. Kim, T., An, J., Jang, J. and Chang, I. (2015). Coupling of anaerobic digester and microbial fuel cell for COD removal and ammonia recovery. *Bioresource Technology*, 195, 217-222.

6. Sotres, A., Cerrillo, M., Viñas, M. and Bonmatí, A. (2015). Nitrogen removal in a two-chambered microbial fuel cell: Establishment of a nitrifying–denitrifying microbial community on an intermittent aerated cathode. *Chemical Engineering Journal*, 284, 905-916.

7. Bock, G. (2016). Removal of High and Low Levels of Ammonium from Industrial Wastewaters. University of Nevada, Las Vegas, UNLV Theses, Dissertations, Professional Papers, and Capstones. 2642, Master of Science in Engineering, Department of Civil and Environmental Engineering and Construction, <http://dx.doi.org/10.34917/9112034>.

8. Sica, M., Duta, A., Teodosiu, C., & Draghici, C. (2014). Thermodynamic and kinetic study on ammonium removal from a synthetic water solution using ion exchange resin. *Clean Technologies and Environmental Policy*, *16*(2), 351-359.

9. Carrera, J., Baeza, J. A., Vicent, T., & Lafuente, J. (2003). Biological nitrogen removal of high-strength ammonium industrial wastewater with two-sludge system. *Water Research*, 37(17), 4211-4221.

10. Gabarró, J., Ganigué, R., Gich, F., Ruscalleda, M., Balaguer, M. D., & Colprim, J. (2012). Effect of temperature on AOB activity of a partial nitritation SBR treating landfill leachate with extremely high nitrogen concentration. *Bioresource technology*, *126*, 283-289.

11. Kinidi, L., Tan, I. A. W., Abdul Wahab, N. B., Tamrin, K. F. B., Hipolito, C. N., & Salleh, S. F. (2018). Recent development in ammonia stripping process for industrial wastewater treatment. *International Journal of Chemical Engineering*, Vol 2018. Article ID 3181087, 14 pages

12. Cui, P., Mai, Z., Yang, S., & Qian, Y. (2017). Integrated treatment processes for coal-gasification wastewater with high concentration of phenol and ammonia. *Journal of Cleaner Production*, *142*, 2218-2226.

13. Johns, M. R. (1995). Developments in wastewater treatment in the meat processing industry: A review. *Bioresource technology*, *54*(3), 203-216.

14. Muyzer, G., De Waal, E. C., & Uitterlinden, A. G. (1993). Profiling of complex microbial populations by denaturing gradient gel electrophoresis analysis of polymerase chain reaction-amplified genes coding for 16S rRNA. Applied and environmental microbiology, 59(3), 695-700.

15. Takai, K. E. N., & Horikoshi, K. (2000). Rapid detection and quantification of members of the archaeal community by quantitative PCR using fluorogenic probes. Applied and environmental microbiology, 66(11), 5066-5072.

16. Jackson, C. R., Langner, H. W., Donahoe‐Christiansen, J., Inskeep, W. P., & McDermott, T. R. (2001). Molecular analysis of microbial community structure in an arsenite‐oxidizing acidic thermal spring. Environmental Microbiology, 3(8), 532-542.

17. J Einen, IH Thorseth, L Øvreås. Enumeration of Archaea and Bacteria in seafloor basalt using real-time quantitative PCR and fluorescence microscopy. *FEMS Microbiology Letters*, Volume 282, Issue 2, May 2008, Pages 182–187, https://doi.org/10.1111/j.1574-6968.2008.01119.x

18. JG Caporaso, CL Lauber, WA Walters, D Berg-Lyons, CA Lozupone, PJ Turnbaugh, N Fierer, R Knight. Global patterns of 16S rRNA diversity at a depth of millions of sequences per sample. *Proc Natl Acad Sci USA*, 2011;15;108:4516-22. doi: 10.1073/pnas.1000080107
